# The Distribution of Phytoecdysteroids among Terrestrial Vascular Plants: A Comparison of Two Databases and Discussion of the Implications for Plant/Insect Interactions and Plant Protection

**DOI:** 10.3390/plants12040776

**Published:** 2023-02-09

**Authors:** Laurence Dinan, Françoise Lafont, René Lafont

**Affiliations:** BIOSIPE, Sorbonne University, Campus Pierre & Marie Curie, 4 Place Jussieu, 75005 Paris, France

**Keywords:** molecular systematics, chemotaxonomy, ecdysteroids, ecdysone, 20-hydroxecdysone, Ecdybase

## Abstract

Phytoecdysteroids are a class of plant secondary compounds which are present in a wide diversity of vascular plant species, where they contribute to a reduction in invertebrate predation. Over the past 55 years, a significant body of heterogeneous literature on the presence, identities and/or quantities of ecdysteroids in plant species has accumulated, resulting in the compilation of a first database, the Ecdybase Literature Survey (ELS; 4908 entries, covering 2842 species). A second extensive database on the distribution of ecdysteroids in vascular plants is available as the Exeter Survey (ES; 4540 entries, covering 4155 species), which used standardised extraction and analysis methods to survey seeds/spores. We compare the usefulness of these two databases to provide information on the occurrence of phytoecdysteroids at the order/family levels in relation to the recent molecular classifications of gymnosperms, pteridophytes/lycophytes and angiosperms. The study, in conjunction with the other published literature, provides insights into the distribution of phytoecdysteroids in the plant world, their role in plant protection in nature and their potential future contribution to crop protection. Furthermore, it will assist future investigations in the chemotaxonomy of phytoecdysteroids and other classes of plant secondary compounds.

## 1. Introduction

There are an estimated 300,000–400,000 species of terrestrial vascular plants on Earth, with about 2000 being discovered each year [1,2]. They are organised into flowerless, seed-producing plants (gymnosperms), ferns (pteridophytes), fern allies (lycophytes) and flowering plants (angiosperms), with 990, 10,578, 1338 and ca. 295,000 species, respectively [1]. The vascular plants are organised into 452 families and 13,467 genera [1]. Plants are continuously subjected to predation and have developed a sophisticated array of physical and chemical strategies to defend themselves, including a diverse range of secondary compounds acting as antifeedants or toxins to potential predators [3].

Phytoecdysteroids are analogues of arthropod steroid hormones, first identified from plant sources by several research groups almost simultaneously in 1966–7 [4,5,6,7,8,9,10]. It became quite rapidly clear that phytoecdysteroids are fairly widely distributed in the plant world, initially in ferns and gymnosperms and then more widely in angiosperms. There have additionally been occasional reports of ecdysteroid and ecdysteroid-like compounds from algae [11,12,13]. Additionally, it was recognised that parts of certain plant species accumulate very high concentrations of ecdysteroids (e.g., 3.2% of the dry weight in stems of *Diploclisia glaucescens* [14] and up to 5% of the dry weight in the roots of *Cyanotis arachnoidea* [15]), while even more customary levels (0.1–1%) are at least 1000-fold higher than the levels found in arthropods. The most commonly encountered ecdysteroid in plants is 20-hydroxyecdysone (20E), which is the major hormonal ecdysteroid in insects, but many other analogues have also been identified, some of which have also been identified from invertebrates, but most of which are currently unique to plants. A compilation of physico-chemical and biological activity data for all (554 as of the 1 December 2022) currently known natural ecdysteroids exists (Ecdybase; Ref. [16]). The ecdysteroid profile of a plant can vary from simple, where one or two analogues are present, to more complex, where major ecdysteroids are accompanied by some minor analogues to form a complex cocktail made up of many different analogues [17,18]. The analogues differ in the number of C-atoms in the side chain, and in the number and location of functional groups and conjugating moieties (Lafont et al., 2002).

The original function attributed to phytoecdysteroids was in contributing to the deterrence of insect predators [5]. Several other hypotheses have also been put forward, e.g., a cell proliferation role in the plant or a sterol storage form [19], but evidence for these or for a plant hormonal role have remained scarce [20]. On the other hand, evidence for the deterrence role, either as antifeedants or hormonal disruptors (reviewed in [21,22]) has steadily accumulated over the years, such that this is now generally accepted, even if it is not as yet fully proven.

The original motivation for investigating a wider range of plant species was to identify high accumulators (>1% of the d.w. as ecdysteroids) and new ecdysteroid analogues. More recently, several studies have begun to assess the chemotaxonomical significance of phytoecdysteroids in the Chenopodiaceae (now part of the Amaranthaceae; Ref. [23]) and the genus *Silene* (Caryophyllaceae; Ref. [24]) and these have revealed useful but complex patterns of distribution. With the advent of molecular classifications for gymnosperms [25], pteridophytes and lycophytes [26] and angiosperms [27] which has reorganised the family structure and identified a linear sequence number (LSN; evolutionary sequence of taxa) for each, we felt that the time was right to examine the distribution of the presence of phytoecdysteroids among vascular plants in a more extensive way. Here, we compare the use of two existing large databases for this purpose. The aim of this article is to bring out the advantages and disadvantages of these two databases for this purpose, and to provide a critical basis for future studies, not only on phytoecdysteroids, but also perhaps for other classes of plant secondary compounds. The study also provides insights into the overall distribution of phytoecdysteroids in the plant world, which plants might be expected to use in their defence strategies against invertebrate predators and which might offer the potential for enhancing ecdysteroid levels/profiles in crop species in order to increase their resistance towards insect and/or nematode pest species.

## 2. Comparison of the Two Databases

### 2.1. General

**Figure 1:**
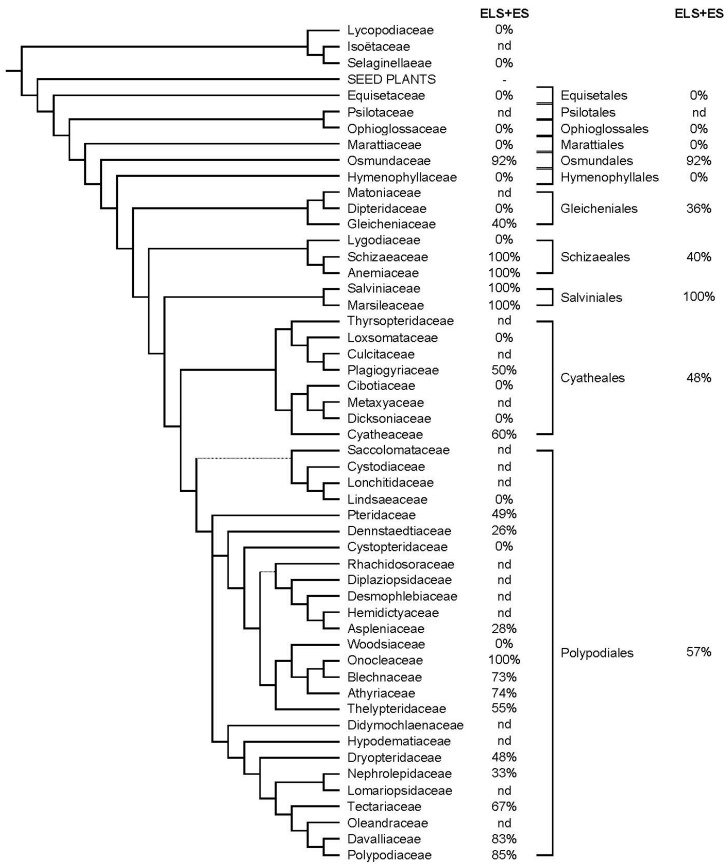
**Lycophytes and pteridophytes:** Indication of the frequency of ecdysteroid-containing species within the orders of lycophytes and pteridophytes. The classification is according to PPG1 (Ref. [26]) and the data concerning the presence or absence of phytoecdysteroids were combined from the ‘Compilation of the literature reports for the screening of vascular plants, algae, fungi and non-arthropod invertebrates for the presence of ecdysteroids’ (ELS Version 11; www.ecdybase.org (accessed on 13 October 2021)), which provides data on 488 lycophyte and pteridophyte species, and the Exeter Survey (ES Version 1: www.ecdybase.org (accessed on 13 October 2021)), which provides data for only 17 pteridophyte species (Table S1). The data are qualitative (i.e., present or absent) and do not necessarily reflect the occurrence of high accumulators. The % frequencies of ecdysteroid-containing species amongst the assessed species in each family and order are indicated in the right-hand columns; nd = no data available.

**Figure 2:**
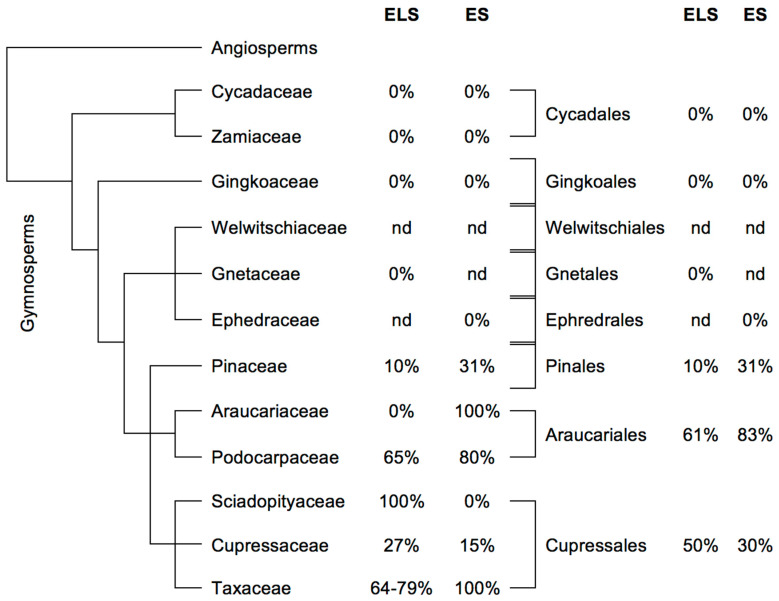
**Gymnosperms:** Indication of the frequency of ecdysteroid-containing species within the orders of gymnosperm plants. The classification is according to Christenhusz et al. [28] and the data concerning the presence or absence of phytoecdysteroids were taken from the ‘Compilation of the literature reports for the screening of vascular plants, algae, fungi and non-arthropod invertebrates for the presence of ecdysteroids’ (ELS Version 11; www.ecdybase.org (accessed on 4 October 2021)), which provides data on 89 gymnosperm species (Table S2), and the Exeter Survey (ES Version 1: www.ecdybase.org (accessed on 4 October 2021)), which provides data on 86 gymnosperm species (Table S3). The data are qualitative (i.e., present or absent) and do not necessarily reflect the occurrence of high accumulators. The % frequencies of ecdysteroid-containing species amongst the assessed species in each order are indicated in the right-hand column; nd = no data available.

**Figure 3:**
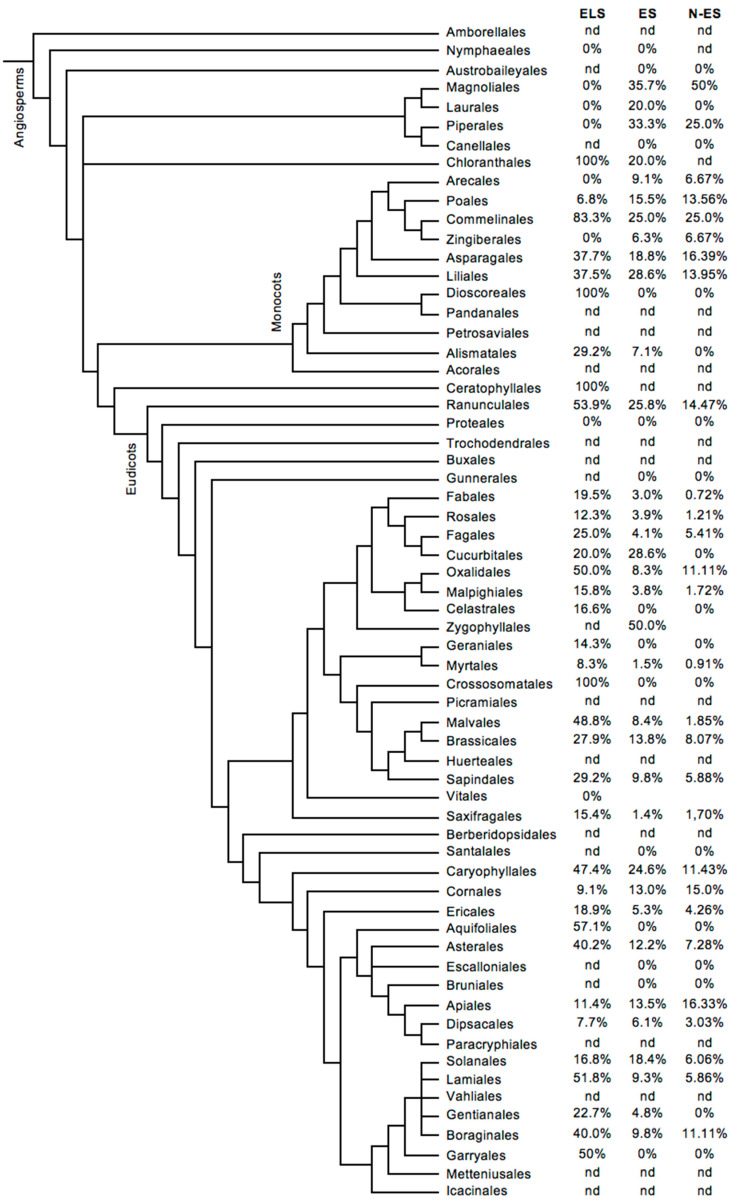
**Angiosperms:** Indication of the frequency of ecdysteroid-containing species within the orders of angiosperm plants. The classification is according to APGIV [27] and the data concerning the presence or absence of phytoecdysteroids were taken from (ELS) the ‘Compilation of the literature reports for the screening of vascular plants, algae, fungi and non-arthropod invertebrates for the presence of ecdysteroids’ (Version 11; www.ecdybase.org (accessed on 8 November 2021)), which provides data on 2282 angiosperm species (Table S4), the Exeter Survey (ES) (Version 1: www.ecdybase.org (accessed on 8 November 2021)), which provides data on 4052 angiosperm species (Table S5) and the randomly selected species of the Exeter survey (N-Series) (N-ES) comprising 2290 angiosperm species (Table S6). The data are qualitative (i.e., present or absent) and do not necessarily reflect the occurrence of high accumulators. The % frequencies of ecdysteroid-containing species amongst the assessed species in each order are indicated in the right-hand column; nd = no data available.

The data concerning ecdysteroid presence at family and order levels are presented in the Supplementary Information in Table S1 for pteridophytes and lycophytes (ELS and ES combined), and Tables S2 (ELS) and S3 (ES) for gymnosperms and Tables S4 (ELS) and S5 (ES) for angiosperms. Table S6 presents the data for the randomly selected ES angiosperm seed samples (N-Series). Table 1 indicates the proportions of species assessed in the two databases for the gymnosperms, lycophytes, pteridophytes and angiosperms. Figure 1, Figure 2 and Figure 3 summarise in diagrammatic form the occurrence of ecdysteroids in relation to the families in the taxonomic trees of the three divisions. Table 2 summarises the distributions of various classes of plant secondary metabolites.

Neither database was generated for the specific purpose of determining the chemotaxonomic significance of phytoecdysteroids. Rather, ELS was compiled as a resource to provide researchers with ready access to the literature, while ES was the product of a survey of plant species to identify those which contained high levels of ecdysteroid agonists (not necessarily steroidal) or ecdysteroid antagonists. However, both databases are large in comparison to those which exist for other classes of plant secondary compounds, even if they are still small in comparison to the total number of vascular plant species (estimated at 400,000). These findings concerning phytoecdysteroids deriving from the two databases will help to refine future studies on the occurrence of phytoecdysteroids in the plant world, and may have relevance to other classes of secondary compounds, especially triterpenoids (bufadienolides, cardenolides, cucurbitacins, saponins and withanolides) which are generated by similar biosynthetic routes and may have parts of their biosynthetic pathways in common.

### 2.2. Sources of the Data

ELS reflects a large body of research performed over more than 50 years by a significant number of researchers of many disciplines with diverse goals. Over this time, the methods of extraction, detection, identification and quantification of ecdysteroids became more sensitive and refined. Many of the early studies were undertaken by phytochemists who isolated individual ecdysteroid analogues and identified them through diligent and time-consuming studies using the nascent spectroscopic methods available at the time. The advent of HPLC facilitated the purification of ecdysteroid analogues enormously, and also allowed their identification via co-chromatography and quantification based on their characteristic UV-absorbance, but in light of what we now know about the complexity of plant secondary metabolites, this may not have been specific enough for unambiguous identification since most were performed using only one separation system (usually C_18_-RP-HPLC). The most recent data are derived from methods which are both sensitive and specific, e.g., HPLC/DAD/MS and advanced NMR techniques. Furthermore, the reports incorporated into ELS concern all or various parts of the plants at diverse stages of development, sometimes not always specified in the publications. Thus, the studies used to compile ELS are very heterogeneous, but the database does accurately reflect the published knowledge. Finally, it should be mentioned that the tendency to publish positive data (the presence of ecdysteroids) over negative findings (the absence of ecdysteroids) will have inevitably biased the content of ELS.

ES reflects the application of a consistent micro-extraction and partial purification strategy and coherent sensitive and specific methods for the detection of ecdysteroids in seed (spores in the case of ferns) samples. The uniformity of the methodology is generally beneficial, but one could argue that other stages of development might vary in their ecdysteroid content. Since the presence of phytoecdysteroids was detected by three ecdysteroid-specific RIAs and the agonist version of the BII cell bioassay, there is the capacity for detection on the basis of chemical similarity to ecdysone (black and white antisera) or 20-hydroxyecdysone (DBL-1 antiserum) and similar biological activity to 20-hydroxyecdysone (BII bioassay). The requirement for linearity in RIA response and the microscopical study of responding BII cells excludes a cytotoxic response and minimises false positives.

The classification of ecdysteroid occurrence in this study is binary (present or absent), i.e., above or below the relevant detection threshold. This is more of a problem for ELS, where a range of methods with differing detection modalities and sensitivities were used, and this may contribute to the larger number of ‘uncertain’ species in the ELS database. However, as phytoecdysteroids are believed to contribute to a reduction in invertebrate predation, the biologically valid threshold concentration would be the level at which predators are deterred, but this would depend on ecdysteroid profile, predator (perhaps several species) and any synergistic interaction with other plant secondary compounds and would thus be ecosystem-specific, so not useful for setting a universal threshold as required for this study.

The proportion of an uncertain presence or absence of ecdysteroids in certain species is higher in the ELS than ES. This refers to situations where different samples of the same species have been assessed either within the same study or different studies and have been found to be contradictory. As mentioned above, this will in part be a result of the differing detection thresholds, but may also arise from different plant parts being compared. In the particular case of ferns, where ‘uncertain’ species are particularly common (Table S1), most of these can be traced back to two major early studies [38,39] where multiple samples of the same species were collected from different locations; some of these were positive, while others were negative. In these cases, the same method was used within each study, so the detection threshold will have been the same. The same parts of the plants (fronds) were compared and misidentification of the plant material seems unlikely. Thus, the most probable explanation would appear to be the existence of ecotypes of these species, which differ in their ecdysteroid content.

A previous comparison using the ES methodology for 180 species of the levels of phytoecdysteroids present in seeds and plants grown from these batches of seeds [40] found that 8.9% of the seed extracts were ecdysteroid-positive, while ecdysteroids were detectable in more of the leaf (48%), stem (16%), root (17%) and flower (33%) extracts. However, only in the four species of which the seeds were strongly positive for ecdysteroids (>1 μg ecdysone eq./g) were the leaves also strongly positive. This study concluded that there was no consistent pattern in the distribution within plants, as it varied from species to species. However, the presence of high levels of ecdysteroids in the seeds does appear to correlate with significant levels in other organs during development, while low or no ecdysteroids in the seeds can be associated with low levels in the leaves. It remains to be assessed whether the levels in species which possess low levels in the leaves are inducible by invertebrate predation or other stressors, but it is our working hypothesis that plants may be ecdysteroid-rich where the compounds are immediately available for defensive purposes, ecdysteroid-negative under all circumstances, where the deterrence of phytophagous invertebrates is achieved by other means, or low ecdysteroid-containing, where the levels are too low for immediate defence, but the existing low level synthesis of ecdysteroids allows for rapid activation and accumulation upon induction by predation [41].

### 2.3. Coverage

#### 2.3.1. Lycophytes and Pteridophytes

Overall, 1.9% of the Lycophyte species have been assessed, but one of the four families, the Isoetaceae, has not been represented. The Equisetaceae (27%) have been overrepresented (Table S1). The percentage of the pteridophyte species that has been assessed in the combined databases is 4.4%. However, 16 of the 47 families have not been represented. Most of these correspond to families with few genera and species, but the Lomariopsidaceae (four genera with 69 species) is a significant omission (Table S1).

#### 2.3.2. Gymnosperms

Two families (Welwitschiaceae and Ephedraceae) are not represented in the ELS (Table S2), while members of the Welwitschiaceae and Gnetaceae are not assessed in the ES (Table S3). On the other hand, the Podocarpaceae and Taxaceae are well represented in the ELS, and the Pinaceae and the Cupressaceae are well represented in the ES. The % overall coverage of the gymnosperms is very similar in the two databases (9.0% in the ELS and 8.7% in the ES; Tables S2 and S3).

#### 2.3.3. Angiosperms

The ELS contains no data for 21 of the 64 angiosperm orders, while this is reduced to 13 for the ES, almost certainly because of the larger size of the latter database. Most correspond to the smaller orders with less than 150 species, but the Dilleniales (430 species), Pandanales (1610 species) and Santales (2373 species) are not represented in the ELS, while the Pandanales is the only larger order not represented in the ES, although the Dilleniales and Santales are still underrepresented. Overall, 0.77% and 1.37% of angiosperm species are covered in the ELS and ES, respectively (Table 1). Amongst the larger orders, the Arecales, Laurales, Myrtales, Piperales and Santales are majorly underrepresented in the ELS, while this can be said of the Laurales, Piperales and Santales in the ES. On the other hand, the Caryophyllales, Commelinales, Liliales and Ranunculales are overrepresented in the ELS, while it is the Liliales and Ranunculales in the ES, but to a lesser extent (Tables S4 and S5). In the cases of the Caryophyllales and Commelinales, this can almost certainly be accounted for by the early discovery of high-accumulating species in these orders (e.g., *Cyanotis arachnoidea* [15] and *Silene otites* [42]), which resulted in a focussed research effort on other closely related species to find other high accumulators.

### 2.4. Taxonomic Distribution of Phytoecdysteroids

#### 2.4.1. Lycophytes and Pteridophytes

The findings indicate that Lycophytes are ecdysteroid-negative, although it should be borne in mind that there are no data for the Isoetaceae (Table S1 and Figure 1). Amongst the early evolving ferns, the Osmundaceae and the Gleicheniaceae contain high proportions of ecdysteroid-positive species, while the other families appear to be ecdysteroid-negative. The families of the later orders (where data are available) appear to be ecdysteroid-negative or contain a significant proportion of ecdysteroid-positive species (Table S1 and Figure 1).

#### 2.4.2. Gymnosperms

The earlier evolving families (Cycadaceae, Zamiaceae, Ginkgoaceae, Gnetaceae and Ephedraceae; no data for the single species in the Welwitschiaceae) all appear to be ecdysteroid-negative, while the Pinaceae, Podocarpaceae, Cupressaceae and Taxaceae amongst the later evolving gymnosperms contain significant proportions of ecdysteroid-positive species. The data for the single-species Sciadopityaceae are contradictory for the two databases (Tables S2 and S3 and Figure 2).

#### 2.4.3. Angiosperms

The proportions of the assessed species found to be ecdysteroid-positive in each angiosperm order according to the ELS and ES are summarised numerically and visually in Figure 3A,B, respectively. The full data are presented in Tables S4 and S5. Overall, 35.9% and 12.3% of the assessed angiosperm species were found to be ecdysteroid-positive in the ELS and ES, respectively. The data from the ELS show that there is a reasonable to good chance (>20%) of encountering ecdysteroid-positive species in 23 of the 43 orders for which there are data, while this is 8 orders from the 51 orders for which there are data for the ES. It is important to bear in mind that this reflects the presence of ecdysteroids at any level, since high accumulators (>0.1% of the dry weight as 20E) are much rarer. The apparently higher probability in the ELS certainly reflects the biases arising from the preferential publication of positive findings and researchers conducting subsequent research on species closely related to those already found to be positive. Both of these biases are significantly reduced in the ES because all results, whether positive or negative, are included in the database and a significant proportion of the samples were randomly selected.

The ELS database suggests that the early evolved orders up to and including the Magnolids are ecdysteroid-negative, even if this is based on evidence from few species. However, the somewhat more extensive ES data show that this is not true, as positive species are present in all three orders of the Magnolids (Piperales, Magnoliales and Laurales). In addition to the numbers of assessed species, other explanations may be greater sensitivity of the methods used in the ES or the presence of detectable ecdysteroids only in seeds of these orders and not at other stages of development (assessed in the ELS, but not in the ES).

From the data for the larger later evolving orders where a greater number of species have been assessed, it would appear that species can always be encountered which are ecdysteroid-positive. A possible exception to this is the Proteales, which might infer a secondary loss of the capacity to biosynthesise/accumulate ecdysteroids in this order, but this would need much more extensive investigation.

#### 2.4.4. Estimate of the Overall % Occurrence of Ecdysteroids in Terrestrial Vascular Plants

The percentage of ecdysteroid-positive seed extracts of randomly selected vascular plant species (the N-series; 2454 species) was 7.1%, which is close to the preliminary value (6.6%) determined previously for 98 species [43], but which owing to the larger number of species involved will be a more reliable estimate for the occurrence of phytoecdysteroids in the seeds of vascular plants. For the seeds of randomly selected angiosperms (2290 species; Table S6), the occurrence of ecdysteroid-positive species is 6.7%.

## 3. Discussion of the Implications for Plant/Insect Interactions and Plant Protection

### 3.1. Co-Evolution of Plants and Insects

It is a widely accepted concept that the co-evolution of plants and insects has driven the wide diversity in both, since vascular plants and herbivorous insects account for more than half of all of the described species, but it has proved difficult to demonstrate that plant secondary compounds and other resistance mechanisms evolved as defensive functions [44]. Since secondary metabolites are not necessary for essential (primary) processes in the plant, they are relatively free to diversify [45] and there is no doubt that vascular plants contain an astounding array of chemically diverse secondary compounds [46]. Several models have been proposed to explain the diversity in plant secondary metabolites (e.g., Refs. [45,47]). The evolution and role of plant chemicals in the co-evolution of plants and insects have been reviewed and discussed [28,29,48,49]. Several possible modes of co-evolution have been recognised, but the ‘escape-and-radiate’ and ‘arms race’ hypotheses, originally proposed, respectively, by Ehrlich and Raven [48] and Dawkins and Krebs [50], seem to be relevant to most (but not all) plant/phytophagous insect interactions. One of the main tenets of plant/insect co-evolution is that that phylogenetic and biochemical diversification has been driven by host shifts through colonisation and specialisation, such that related phytophagous insects feed on related plant species [51], and much more rarely on unrelated plant species, presumably because other attraction/deterrence features (including nutritional suitability) are more likely to be shared by related plant species. The model proposes that the plant develops a new or better defence which deters current predator(s) and improves the plant’s viability and fitness, allowing it to radiate. This increased abundance makes the plant more attractive to other potential predators, which are able to overcome the new defence. Thus, the co-evolution is decoupled, such that the radiation of the adapted insects necessarily post-dates the radiation of the modified plants [44]. The accumulation of genetic changes ultimately results in increased speciation and diversity. It is probable that most defence strategies are ‘diffuse’, i.e., they operate against a guild of insect species (which may or may not be related), rather than against a single species, as individuals of a single insect species will rarely be numerous enough to exert selection pressure at the population level of the plant species. However, monophagous and oligophagous predators may defoliate individual plants and exert high herbivore pressure at the level of the individual plant and thus promote dissimilar plant defensive chemistry and specialisation, whereas polyphagous species exert lower herbivore pressure and may permit the evolution of more chemically similar plants and generalisation [52]. In some cases, the compounds which deter non-adapted insects may come to act as feeding or oviposition stimulants to insects which have come to specialise on the plant (e.g., cucurbitacins in various members of the Cucurbitaceae for diabrotica beetles [53]).

The first fossil plants and fungi have been dated to 480–460 mya. Arthropods began to invade the land in the Silurian period (440 mya) when land plants comprised mosses and lycophytes. The divergence in the ferns (400 mya) occurred at the start of the Devonian period as the first winged insects appeared, but many of the modern ferns did not appear until ca. 145 mya. Important orders of phytophagous insects, Orthoptera, Hemiptera, Coleoptera, Lepidoptera and Diptera emerged 310–240 mya [54]. The divergence and spread of the gymnosperms (350 mya) coincided in the Carboniferous period with the first great radiation of insects and that of the angiosperms (150 mya) in the Cretaceous period with the second great radiation of insects. Insects and plants may interact to mutual benefit (in pollination, protection and seed dispersal [55]) and this appears to be an ancient form of insect–plant interaction since some gymnosperms (cycads and gnetophytes) are primarily insect-pollinated. The rapid diversification during the Cretaceous period and ultimate dominance of the angiosperms have been ascribed to animal-mediated pollination, after the emergence of the Hymenoptera (250–200 mya [54]). However, most insect–plant interactions (phytophagous predation) have the potential to be seriously detrimental to the plant, such that sessile plants need to develop effective and sophisticated ways of defending themselves. The first secondary metabolites were produced by aquatic organisms and an impressive increase in biochemical diversity occurred in vascular plants during the Devonian and early Carboniferous periods, resulting in the formation of lignin derivatives, tannins, terpenoids, alkaloids, flavonoids, etc. (Refs. [3,28], Table 2). Secondary metabolites involved in resistance can be constitutive or induced. Furthermore, the defences can be ‘quantitative’ (reducing digestibility) or ‘qualitative’ (having toxic effects), and it has been suggested that plants which are fixed in space and time (e.g., trees) should possess quantitative defences whereas plants without predictable distributions (e.g., herbaceous plants) should produce qualitative defences [28]. Elaborating the analogues of the insects’ steroid hormones as protective chemicals (as antifeedants or endocrine disruptors) would, in turn, promote evasive strategies in the predatory insects (rapid metabolism/excretion of ingested ecdysteroids or perception of plant ecdysteroids via taste receptors), leading to the further refinement of plant defence strategies (e.g., modification of ecdysteroid profile or varying concentrations within the plant or at different developmental stages). Thus, insects will have been a major driving force in the evolution and diversification of terrestrial vascular plants, while plants will have promoted the diversification and radiation of insects, in part mediated by phytoecdysteroids.

### 3.2. Implications for Plant Protection

The topic of ecdysteroids as defensive compounds in plants and animals has recently been extensively reviewed [56]. In plants, evidence is accumulating that phytoecdysteroids serve as deterrents of feeding or egg deposition perceived by taste receptors on the potential predator’s mouth parts or legs, respectively, or as endocrine disruptors on ingestion by susceptible insect or nematode species. Although 20-hydroxyecdysone (the major hormonal ecdysteroid in insects) remains the most frequently encountered phytoecdysteroid, the situation is complex and reflects the competition between plants and phytophagous invertebrates for the upper hand, since certain insects are partially or fully resistant to dietary 20-hydroxyecdysone either through effective detoxification reactions to generate hormonally less-active metabolites or by confining 20-hyroxyecdysone to the gut lumen (i.e., exceedingly low bioavailability) by an unknown mechanism together with rapid excretion in the faeces. Some of the detoxification methods are specific to certain classes of ecdysteroid (e.g., conjugation or acylation of 22-hydroxyecdysteroids) and these can be circumvented by feeding 22-deoxyecdysteroids, which although hormonally less active than 20E are more active than 22-glycosylated or 22-acylated ecdysteroids, as has been demonstrated for ticks [57]. This goes some way to provide a reasonable explanation for the large number of phytoecdysteroid analogues and the diversity in ecdysteroid levels/profiles found in phytoecdysteroid-containing plants, since the total levels of ecdysteroids can be expected to be related to the susceptibility of potential predators, and the complexity of the ecdysteroid profile provides flexibility to circumvent the present or future detoxification mechanisms in the predators. Non-polar conjugated phytoecdysteroids (e.g., benzoates, cinnamates), which would be expected to have low hormonal activity per se, may have higher bioavailability and then be hydrolysed within the predator to release active endocrine disruptor. It should be borne in mind that phytoecdysteroids should not be considered in isolation, since they may act in synergy with other types of plant secondary compounds to provide an overall effective protection system. An example of one such plant is common bracken (*Pteridium aquilinum*), of which the mature fronds are highly resistant to predators of all sorts (invertebrate and vertebrate), and which contains a wide variety of defensive chemicals (including phytoecdysteroids; Ref. [8]), but each at relatively low levels [58,59,60]. *Pteridium* is one of the oldest genera of ferns with a fossil record which goes back more than 55 million years [61]. The ELS database, which, in itself, gives qualitative information about the presence of Phytoecdysteroids, can be mined to examine the original literature to determine whether further information is available on ecdysteroid levels and profiles. The ES database already provides more quantitative values and agonist and antagonist activity data for extracts. Furthermore, all positive extracts were analysed via RP- and NP-HPLC coupled with RIA and BII cell bioassay to provide data on ecdysteroid profiles. Together with the known information on the invertebrates associating with the plant by using it as a host plant (e.g., HOSTS, Ref. [62]; DBIF, Ref. [63]) or by being a serious predator (CAB Direct, Ref. [64]), this provides a good starting point for considering the raison d’être for the presence and function of ecdysteroids in the plant and developing relevant hypotheses on the role of phytoecdysteroids in the interaction.

### 3.3. Prospects and Applications

One of the early goals in phytoecdysteroid research was to identify plant species which are high accumulators of 20-hydroxyecdysone and other analogues. These high accumulators were, and still are, our main source of pure ecdysteroids for studies in invertebrate physiology/biochemistry and the wide diversity in ecdysteroid analogues available from plant sources has proved to be an exceedingly useful natural resource for structure–activity relationship studies. Relating the occurrence of phytoecdysteroids to molecular phylogenetic trees will facilitate a more rational search for high accumulators. This search has recently received greater interest because of the pharmaceutical/medical potential of ecdysteroids for the treatment of various conditions in mammals (including humans). Ecdysteroids are non-toxic to mammals and were shown to have many beneficial effects in model animal systems. Recently, clinical trials have started to substantiate these findings. For such trials to proceed, kg amounts of pure 20-hydroxyecdysone are required, and for ultimate development as a commercial medication, tonnes would be needed. The only currently viable source of such large amounts of 20E is from high accumulating plants, since other methods (e.g., chemical synthesis) are too inefficient (reviewed in [65]).

Since less than 2% of the known species of vascular plants have been investigated at any stage of development, there is plenty of scope to expand this study. This report, which pulls together all of the currently known surveys of the presence of phytoecdysteroids, reveals that the coverage is not fully satisfactory even for the consideration of the distribution at the order level, since some orders are underrepresented or not represented at all. Much more extensive studies (covering 20–25,000 species) could only be envisaged as major international collaborations where botanists in different parts of the world collect and identify many samples from their local flora and these are analysed using standardised and optimised analytical procedures. A start in this direction has been made for the floras of north-east European Russia and northern Vietnam [66,67]. It would be desirable to know more about the distribution of phytoecdysteroids at the family and genus levels, but this could only be feasible for large and extensively studied groups as and when molecular systematic data at the sub-order level become available.

A further advance in this area would be to improve a high accumulator species so that it produces even larger amounts of 20-hydroxyecdysone and possesses a simpler ecdysteroid profile, which would considerably facilitate the purification of 20E considerably. Progress is currently hampered by our lack of knowledge of the biosynthetic pathway(s) for phytoecdysteroids and their regulation. What information we have about the biosynthesis is incomplete and derived from only a few species, but intriguingly suggests that the routes may be different in ferns and flowering plants. The data here confirm that phytoecdysteroids are widespread in pteridophytes, gymnosperms and angiosperms, but indicate that they are absent in earlier evolving lycophytes (fern allies). Moreover, within the gymnosperms and angiosperms, there is some evidence to suggest that ecdysteroids are absent in the earlier evolving orders. The distribution pattern seen in Figure 3 might also suggest that ecdysteroid accumulation is polyphyletic, which is supported by evidence that the early stages of ecdysteroid biosynthesis are different in ferns and angiosperms (reviewed in [21]), as suggested by Zhang et al. [68] for a range of secondary metabolites in plants. Other lines of evidence [40] indicate that most, if not all, vascular plants have the genetic capacity to produce phytoecdysteroids, which might favour a monophyletic origin. It is feasible that the presence of the genes for ecdysteroid biosynthesis is monophyletic, while the regulation of their expression is polyphyletic. Currently, the evidence is just too sparse to be able to decide between mono- and polyphyletic, but future molecular systematic studies together with molecular and biochemical research aimed at elucidating the phytoecdysteroid biosynthetic pathway(s) and their regulation will help to resolve this.

### 3.4. Possible Relevance to Other Classes of Triterpenoids

Table 2 summarises the known distributions of various classes of plant secondary compounds in vascular plants and demonstrates that the different classes have very disparate distributions. This also applies to the triterpenoids, of which the phytoecdysteroids are a constituent class. In contrast to the wide distribution of phytoecdysteroids in plant families, other classes of triterpenoid secondary products (bufadienolides, cardenolides, cucurbitacins and withanolides) are associated with relatively few families each, and, in reality, are restricted to particular sub-families and tribes within these families, which are not necessarily taxonomically close, leading to the proposal that there were multiple origins for each class of secondary metabolite during plant evolution (Ref. [68], which does not specifically consider phytoecdysteroids). A caveat does perhaps need to be mentioned here in that plants in other families may be able to produce low levels of the non-phytoecdysteroid triterpenoids, but they may not have been detectable by the less sensitive detection methods used when compared to the great sensitivity of the immunological methods used for the phytoecdysteroids. However, even if one considers only the species containing more significant amounts of phytoecdysteroids, the range of families containing phytoecdysteroid-positive species is far greater than that of the other classes of triterpenoids. Certain plants accumulate more than one class of triterpenoid (e.g., bufadienolides and ecdysteroids in species of *Helleborus*: [69,70]). Plants biosynthesise triterpenoids through the MVA-pathway, by building them up from C_5_ isopentenyl diphosphate units (from cytosolic mevalonate) to form C_30_ squalene which is then cyclised to generate the characteristic four-ring steroid nucleus and side chain [71,72]. The different classes of plant triterpenoids are derived from cholesterol or a plant sterol (C_27_–C_30_) which is converted in a committed step reaction to a core metabolite which will then be further metabolised via the permutation of modification reactions (oxidations, reduction, hydroxylations, conjugations and epimerisations) to generate the characteristic triterpenoid profile for that plant species at that stage of its development. The types and locations of the various further modifications overlap considerably between the different classes of plant triterpenoids. It appears that these further modifications occur as a network of reactions (rather than a linear sequence), which implies lower substrate specificity of the reaction involved (as proposed by Firn and Jones [47]), but provides greater flexibility in the final product range and profile, which is effectively determined by the flux through the network. One may speculate that if the substrate specificities of the modification enzymes are broad enough that some or all of these enzymes could act on the core metabolites and later intermediates of the various triterpenoid pathways, this would provide the plant with greater genetic efficiency and flexibility to respond to, and resist, predation pressure either via induction within a generation or long-term modification of expressed pathways over generations. Combining this with the hypothesis that most, if not all, plants have the genetic capacity to produce phytoecdysteroids, it seems plausible that phytoecdysteroids were the first class of plant triterpenoids to evolve as defensive compounds, initially as a general defence against insect attack but later as a more modulated specific resistance mechanism against non-adapted invertebrates. The other triterpenoid pathways could then have arisen via gene duplication and modification of the reaction generating the core metabolite to produce the other classes of triterpenoids with different biological activities in invertebrates (e.g., cucurbitacins are deterrents to most insects but are attractants to some specialised adapted insects and operate as ecdysteroid antagonists acting at the insect ecdysteroid receptor [73,74,75]) and deterrent effects on other types of predators (including vertebrates). 

## 4. Materials and Methods

### 4.1. Databases

#### 4.1.1. Ecdybase Literature Survey (ELS)

The ELS (Ref. [56]; continuously updated; the plant part also exists as an Excel file (available upon request from the authors)) is a compilation of the published literature from 1967 to the present day concerning the occurrence of phytoecdysteroids in plants, algae, fungi and non-arthropod invertebrates. The major section (Section 1) concerning reports about vascular plants lists the species, their families and whether ecdysteroids were detected or not, and with the relevant citation and abstract (where available). Version 11 (September 2021) was used for this analysis. It contains information on 2842 species of vascular plants, regardless of the plant part(s) or stage(s) of development indicated in the cited publication. The methods of detection, identification and quantification of phytoecdysteroids vary considerably in the reports from co-chromatography on HPLC or TLC to isolation and unambiguous identification via physico-chemical methods (NMR, MS, etc.) and in sensitivity from fluorescence quenching on TLC plates to ecdysteroid-specific immunoassays and bioassays. The methods of collection, preparation, extraction, identification and quantification are available from the cited literature in the ELS [56].

#### 4.1.2. Exeter Survey (ES)

The ES [76] derives from a 7-year study where standardised methodology was used in a strategy [43] to screen the seeds/spores of 4155 species of vascular plants for the presence of ecdysteroid agonists and antagonists. Samples were micro-extracted with methanol (in which ecdysteroids are highly soluble) and the extracts were partially purified (to remove non-polar lipids and pigments) before analysis with 2 or 3 ecdysteroid-specific radioimmunoassays (using the black, white and DBL-1 antisera to quantify compounds structurally similar to ecdysone and 20-hydroxyecdysone; Refs. [43,77]) and the *Drosophila melanogaster* BII microplate bioassay for ecdysteroid agonists (detecting the presence of compounds with the biological activity of 20-hydroxyecdysone; Ref. [78]). The threshold for the detection of ecdysteroids was 150 ng ecdysone equivalents/g for the black and white antisera, 75 ng ecdysone equivalents/g for the DBL-1 antiserum and 7.2 μg 20-hydroxyecdysone equivalents/g for the BII bioassay. The methods used are described in detail on the website [76], and the data are presented in tabular form. The samples were either randomly selected (N-Series; 2500 species) or selected according to potential interest (S-Series; 2000 species). The criterion for being deemed positive was a linear response (pg ecdysone equivalents vs. extract aliquot size) in at least one RIA or a positive response in the agonist version of the BII bioassay.

### 4.2. Processing of Data

The plant species in the two databases were originally allocated to families according to Brummitt [79], which was an authoritative classification at the time that they were originally compiled. Since then, new classifications based on molecular sequencing data have appeared (PPG1 [26] for ferns and lycophytes, Christenhusz et al. [25] for gymnosperms and APG IV [27] for angiosperms), so the species in the two databases were reclassified according to the new classifications by entering each genus name into Wikipedia to ascertain the present classification. In the process, certain changes in nomenclature at the genus and species level were also detected and incorporated. Subspecies and varieties of a species were treated as samples of that species, and not separately. The tables in the Supplementary Information (Tables S1–S6) were generated by manual tally of the number of species in each family which were ecdysteroid-positive, ecdysteroid-negative or uncertain. For the gymnosperm and angiosperm data, separate tables were prepared for ELS and ES, but for the pteridophytes and lycophytes, not enough data were present in the ES (only 18 entries; see Discussion), so the ELS and ES data were combined to give a single table. The numbers of known plant genera and species in each family were obtained from Christenhusz and Byng [1].

## Figures and Tables

**Table 1:** The proportions of species assessed in the two databases.

		% of All Species Assessed	
	No. Species #	ELS	ES
**Gymnosperms**	990	9.0%	8.7%
**Lycophytes**	1338	1.9% *	
**Pteridophytes**	10,578	4.4% *	
**Angiosperms**	295,383	0.77%	1.37%

* There are too few species present in the ES for meaningful data for ELS and ES to be calculated separately. # from Christenhusz and Byng [1].

**Table 2:** Distribution of the main classes of plant secondary metabolites according to plant families with emphasis on triterpenoids (modified and extended from Kariñho-Betancourt [28]).

Class	Pathway	Plant Families	References
Alkaloids	Shikimic acid pathway	Fabaceae, Liliaceae, Solanaceae, Papaveraceae, Apocynaceae, Amaryllidaceae, Rununculaceae	[29]
Cyanogenic glycosides	Shikimic acid pathway	Most vascular plants: gymnosperms and angiosperms	[3]
Flavonoids	Phenylpropanoid pathway	All plants	[28,30]
Glucosinolates	Shikimic acid pathway	Brassicaceae, Capparidaceae, Tropaolaceae	[3]
Latex and resins	Various pathways	Ca. 10% of angiosperms	[3,28]
Mono- and diterpenes	MEP pathway	Lamiaceae	[29]
Non-protein amino acids	Modified AA pathways and novel metabolic routes	Fabaceae	[29,31]
Phenolics	Shikimic acid and/or malonic acid pathways	Widely distributed in vascular plants	[3]
**Triterpenoids**	MVA pathway		
Bufadienolides		Liliaceae, Crassulaceae, Iridaceae, Melanthiaceae, Ranunculaceae, Santalaceae	[32]
Cardenolides		Apocynaceae, Liliaceae, Ranunculaceae, Moraceae, Fabaceae, Scrophulariaceae, Cruciferae, Sterculaceae, Euphorpiaceae, tiliaceae, Celastraceae	[33,34]
Cucurbitacins		Cucurbitaceae	[35]
Phytoecdysteroids		Wide distribution	This report
Steroidal alkaloids		Buxaceae, Liliaceae, Apocynaceae, Solanaceae	[29]
Steroidal saponins		Monocotoledonous angiosperms (Agavaceae, Alliaceae, Asparagaceae, Dioscoreaceae, Liliaceae, Taccaceae) + Solanaceae	[36]
Triterpenoid saponins		Dicotyledonous angiosperms (Amaranthaceae, Apiaceae, Caryophyllaceae, Fabaceae, Ranunculaceae)	[36]
Withanolides		Mainly from certain genera in the Solanaceae (e.g., Iochroma, Datura, Jabrosa, Physalis, Salpichroa, Withania), but also certain species in the Taccaceae, Fabaceae, Dioscoraceae, Myrtaceae and Lamiaceae	[37]

## Data Availability

The two databases (ELS and ES) are online at www.ecdybase.com.

## Supplementary Materials

The following supporting information can be downloaded at https://www.mdpi.com/article/10.3390/plants12040776/s1: Table S1: The Distribution of Ecdysteroid-Positive Species Within Pteridophyte (Fern) and Lycophyte (Fern Ally) orders; data from the Ecdybase Literature Survey (ELS) and the Exeter Survey (ES); Table S2: The Distribution of Ecdysteroid-Positive Species Within Gymnosperm orders; Data from the Ecdybase Literature Survey (ELS); Table S3: The Distribution of Ecdysteroid-Positive Species Within Gymnosperm orders; Data from the Exeter Survey (ES); Table S4: The Distribution of Ecdysteroid-Positive Species Within Angiosperm orders; Data from the Ecdybase Literature Survey (ELS); Table S5: The Distribution of Ecdysteroid-Positive Species Within Angiosperm orders; Data from the Exeter Survey (ES); Table S6: The Distribution of Ecdysteroid-Positive Species Within Angiosperm orders; Data from the randomly selected samples (N-Series) of the Exeter Survey (ES).
