# Supplementary material for: The Distribution of Phytoecdysteroids among Terrestrial Vascular Plants: A Comparison of Two Databases and Discussion of the Implications for Plant/Insect Interactions and Plant Protection
Source: Plants (Basel). 2023 Feb 9;12(4):776. doi: 10.3390/plants12040776 (PMC9967490; doi:10.3390/plants12040776)
Supplement: Supplementary file 1 [file plants-12-00776-s001.zip › plants-2124931-supplementary.pdf]

**Supplementary information**

# Table S1: The Distribution of Ecdysteroid-Positive Species Within Pteridophyte (Fern) and Lycophyte (Fern-Ally) Orders; data from the Ecdybase Literature Survey (ELS) and the Exeter Survey (ES)

Orders and Families according to PPG1 (J Systematics & Evolution 54(6), 563-603, 2016)

The data on the presence or absence of ecdysteroids derives from the Ecdybase Literature Survey Version 11 (accessed on the 20/08/2021) and the Exter Survey (Version 1) combined.

| Fern/Fern-Ally Order | Families + [linear sequence number] | No. Genera | No. Species | No. Species Assessed | No. Species Positive | No. Species Negative | No. Species Uncertain* | % of All Species Positive | % of Assessed Species Positive |
|---|---|---|---|---|---|---|---|---|---|
| **Fern-Allies** | | | | | | | | | |
| Lycopodiales | Lycopodiaceae [1] | 16 | 388 | 13 (3.4%) | | 12 | 1 | 0% | 0% |
| Isoetales | Isoetaceae [2] | 1 | ca. 250 | 0 (0%) | | | | | |
| Sellaginelles | Sellaginellaceae [3] | 1 | ca. 700 | 9 (1.3%) | | 9 | | 0% | 0% |
| Equisetales | Equisetaceae [4] | 1 | 15 | 4 (26.7%) | | 3 | 1 | 0% | 0% |
| **All Lycophytes** | **4** | **19** | **1338** | **26 (1.9%)** | | **24** | **2** | **0%** | **0%** |
| | | | | | | | | | |
| **Ferns** | | | | | | | | | |
| Psilotales | Psilotaceae [5] | 2 | 17 | 0 (0%) | | | | | |
| Ophioglossales | Ophioglossaceae [6] | 10 | 112 | 5 (4.5%) | | 5 | | 0% | 0% |
| Marattiales | Marattiaceae [7] | 6 | 111 | 2 (1.8%) | | 2 | | 0% | 0% |
| Osmundales | Osmundaceae [8] | 6 | 18 | 12 (66.7%) | 11 | 1 | | 61% | 92% |
| Hymenophyllales | Hymenophyllaceae [9] | 9 | 434 | 14 (3.2%) | | 14 | | 0% | 0% |
| Gleicheniales | Matoniaceae [10]<br>Dipteridaceae [11]<br>Gleicheniaceae [12] | 2<br>2<br>6 | 4<br>11<br>157 | 0 (0%)<br>1 (9.1%)<br>10 (6.4%) | <br><br>4 | <br>1<br>6 | | <br>0%<br>3% | <br>0%<br>40% |
| **All Gleicheniales** | **3** | **10** | **172** | **11 (6.4%)** | **4** | **7** | | **2%** | **36%** |
| Schizaeales | Lygodiaceae [13] | 1 | 40 | 3 (7.5%) | | 2 | 1 | 0% | 0% |

| | | | | | | | | | |
|---|---|---|---|---|---|---|---|---|---|
| | Schizeaceae [14] | 2 | 35 | 1 (2.9%) | 1 | | | 3% | 100% |
| | Anemiaceae [15] | 1 | 115 | 1 (0.9%) | 1 | | | 1% | 100% |
| **All Schizeales** | **3** | **4** | **190** | **5 (2.6%)** | **2** | **2** | **1** | **1%** | **40%** |
| Salviniales | Salviniaceae [16] | 2 | 21 | 2 (9.5%) | 2 | | | 10% | 100% |
| | Marsileaceae [17] | 3 | 61 | 1 (1.6%) | 1 | | | 2% | 100% |
| **All Salviniales** | **2** | **5** | **82** | **3 (3.7%)** | **3** | | | **4%** | **100%** |
| Cyatheales | Thyrsopteridaceae [18] | 1 | 1 | 0 (0%) | | | | | |
| | Loxsomataceae [19] | 2 | 2 | 1 (50.0%) | | 1 | | 0% | 0% |
| | Culcitaceae [20] | 1 | 2 | 0 (0%) | | | | | |
| | Plagiogyriaceae [21] | 1 | 15 | 6 (40.0%) | 3 | 2 | 1 | 20% | 50% |
| | Cibotiaceae [22] | 1 | 9 | 1 (11.1%) | | 1 | | 0% | 0% |
| | Metaxyaceae [23] | 1 | 6 | 0 (0%) | | | | | |
| | Dicksoniaceae [24] | 3 | 35 | 4 (11.4%) | | 4 | | 0% | 0% |
| | Cyatheaceae [25] | 3 | 643 | 15 (2.3%) | 9 | 5 | 1 | 1% | 60% |
| **All Cyatheales** | **8** | **13** | **713** | **25 (3.5%)** | **12** | **13** | **2** | **2%** | **48%** |
| Polypodiales | Saccolomataceae [26] | 1 | 18 | 0 (0%) | | | | | |
| | Cystodiaceae [27] | 1 | 1 | 0 (0%) | | | | | |
| | Lonchitidaceae [28] | 1 | 2 | 0 (0%) | | | | | |
| | Lindsaeaceae [29] | 7 | 234 | 6 (2.6%) | | 6 | | 0% | 0% |
| | Pteridaceae [30] | 53 | 1211 | 53 (4.4%) | 26 | 17 | 10 | 2% | 49% |
| | Dennstaedtiaceae [31] | 10 | 265 | 23 (8.7%) | 6 | 12 | 5 | 2% | 26% |
| | Cystopteridaceae [32] | 3 | 37 | 1 (2.7%) | | 1 | | 0% | 0% |
| | Rachidosoraceae [33] | 1 | 8 | 0 (0%) | | | | | |
| | Diplaziopsidaceae [34] | 2 | 4 | 0 (0%) | | | | | |
| | Desmophlebiaceae [35] | 1 | 2 | 0 (0%) | | | | | |
| | Hemidyctyaceae [36] | 1 | 1 | 0 (0%) | | | | | |
| | Aspleniaceae [37] | 2 | 730 | 29 (4.0%) | 8 | 18 | 3 | 1% | 28% |
| | Woodsiaceae [38] | 1 | 39 | 1 (2.6%) | | 1 | | 0% | 0% |
| | Onocleaceae [39] | 4 | 5 | 5 (100%) | 5 | | | 100% | 100% |
| | Blechnaceae [40] | 24 | 265 | 26 (9.8%) | 19 | 6 | 1 | 7% | 73% |
| | Athyriaceae [41] | 3 | 650 | 50 (7.7%) | 37 | 5 | 8 | 6% | 74% |
| | Thelypteridaceae [42] | 30 | 1034 | 31 (3.0%) | 17 | 10 | 4 | 2% | 55% |
| | Didymochlaenaceae [43] | 1 | 1 | 0 (0%) | | | | | |

| | | | | | | | | | |
|---|---|---|---|---|---|---|---|---|---|
| | Hypodematiaceae [44] | 2 | 22 | 0 (0%) | | | | | |
| | Dryopteridaceae [45] | 26 | 2115 | 88 (4.2%) | 42 | 31 | 15 | 2% | 48% |
| | Nephrolepidaceae [46] | 1 | 19 | 3 (15.8%) | 1 | 2 | | 5% | 33% |
| | Lomariopsidaceae [47] | 4 | 69 | 0 (0%) | | | | | |
| | Tectariaceae [48] | 7 | 250 | 3 (1.2%) | 2 | 1 | | 1% | 67% |
| | Oleandraceae [49] | 1 | 15 | 0 (0%) | | | | | |
| | Davalliaceae [50] | 1 | 65 | 6 (9.2%) | 5 | 1 | | 8% | 83% |
| | Polypodiaceae [51] | 65 | 1652 | 60 (3.6%) | 51 | 5 | 4 | 3% | 85% |
| **All Polypodiales** | **26** | **253** | **8455** | **385 (4.6%)** | **220** | **116** | **50** | **3%** | **57%** |
| **All Pteridophytes** | **47** | **318** | **10,578** | **462 (4.4%)** | **250** | **159** | **53** | **2.4%** | **54.1%** |
| | | | | | | | | | |
| **All Lycophytes and Pteridophytes** | **51** | **337** | **11,916** | **488 (4.1%)** | **250** | **183** | **55** | **2.1%** | **51.1%** |

*The high proportion of 'uncertain' derives from samples of the same species collected from different locations sometimes being positive and sometimes negative (see Hikino H et al. 1973 and Yen KY et al 1974).

The data for only 17 species (from 488) derived from the Exeter Survey, because ferns are underrepresented in the Survey. The reason for this is probably that the samples derived from commercial Seed Suppliers and very few gardeners wish to grow ferns from spores.

# Table S2: The Distribution of Ecdysteroid-Positive Species Within Gymnosperm Orders; Data from the Ecdybase Literature Survey (ELS)

Orders and Families according to Christenhusz MJM et al. (Phytotaxa 19, 55-70, 2011)

The data on the presence or absence of ecdysteroids derives from the Ecdybase Literature Survey Version 11 (accessed on the 20/08/2021)

| Gymnosperm Order | Families + [linear sequence number*] | No. Genera | No. Species | No. Species Assessed | No. Species Positive | No. Species Negative | No. Species Uncertain | % of All Species Positive | % of Assessed Species Positive |
|---|---|---|---|---|---|---|---|---|---|
| Cycadales | Cycadaceae [1]<br>Zamiaceae [2] | 1<br>9 | ca. 107<br>ca. 206 | 1 (0.9%)<br>1 (0.5%) | | 1<br>1 | | 0%<br>0% | 0%<br>0% |
| All Cycadales | | 10 | 313 | 2 (0.6%) | | 2 | | 0% | 0% |
| Ginkgoales | Ginkgoaceae [3] | 1 | 1 | 1 (100%) | | 1 | | 0% | 0% |
| Welwitschales | Welwitschiaceae [4] | 1 | 1 | 0 (0%) | | | | | |
| Gnetales | Gnetaceae [5] | 1 | 30 | 1 (3.3%) | | 1 | | 0% | 0% |
| Ephedrales | Ephedraceae [6] | 1 | ca. 40 | 0 (0%) | | | | | |
| Pinales | Pinaceae [7] | 11 | ca. 225 | 10 (4.4%) | 1 | 9 | | 0.5% | 10% |
| Araucariales | Araucariaceae [8]<br>Podocarpaceae [9] | 3<br>19 | 41<br>ca. 180 | 3 (7.3%)<br>46 (25.6%) | 0<br>30 | 3<br>10 | <br>6 | 0%<br>17% | 0%<br>65% |
| All Araucariales | | 22 | 221 | 49 (22.2%) | 30 | 13 | 6 | 14% | 61% |
| Cupressales | Sciadopityaceae [10]<br>Cupressaceae [11]<br>Taxaceae [12] | 1<br>29<br>6 | 1<br>ca. 130<br>28 | 1 (100%)<br>11 (8.5%)<br>14 (50.0%) | 1<br>3<br>9 | 0<br>8<br>3 | <br><br>2 | 100%<br>2%<br>32-39% | 100%<br>27%<br>64-79% |
| All Cupressales | | 36 | 159 | 26 (16.4%) | 13 | 11 | 2 | 8% | 50% |
| **All Gymnosperms** | | **83** | **990** | **89 (9.0%)** | **44** | **37** | **8** | **4.4-5.3%** | **49-58%** |

# Table S3: The Distribution of Ecdysteroid-Positive Species Within Gymnosperm Orders; Data from the Exeter Survey (ES)

Orders and Families according to Christenhusz MJM et al. (Phytotaxa 19, 55-70, 2011)

Data on the presence/absence of ecdysteroids derives from the Exeter Survey of plants for ecdysteroid agonists and antagonists (1995-2002; Version 1)

| Gymnosperm Order | Families + [linear sequence number*] | No. Genera | No. Species | No. Species Assessed | No. Species Positive | No. Species Negative | No. Species Uncertain | % of All Species Positive | % of Assessed Species Positive |
|---|---|---|---|---|---|---|---|---|---|
| Cycadales | Cycadaceae [1]<br>Zamiaceae [2] | 1<br>9 | ca. 107<br>ca. 206 | 1 (0.9%)<br>4 (1.9%) | | 1<br>4 | | | 0%<br>0% |
| All Cycadales | | 10 | 313 | 5 (1.6%) | | 5 | | | 0% |
| Ginkgoales | Ginkgoaceae [3] | 1 | 1 | 1 (100%) | | 1 | | | 0% |
| Welwitschales | Welwitschiaceae [4] | 1 | 1 | | | | | | - |
| Gnetales | Gnetaceae [5] | 1 | 30 | | | | | | - |
| Ephedrales | Ephedraceae [6] | 1 | ca. 40 | 2 (5.0%) | | 2 | | | 0% |
| Pinales | Pinaceae [7] | 11 | ca. 225 | 39 (17.3%) | 12 | 27 | | | 31% |
| Araucariales | Araucariaceae [8]<br>Podocarpaceae [9] | 3<br>19 | 41<br>ca. 180 | 1 (2.4%)<br>5 (2.8%) | 1<br>4 | <br>1 | | | 100%<br>80% |
| All Araucariales | | 22 | 221 | 6 (2.7%) | 5 | 1 | | | 83% |
| Cupressales | Sciadopityaceae [10]<br>Cupressaceae [11]<br>Taxaceae [12] | 1<br>29<br>6 | 1<br>ca. 130<br>28 | 1 (100%)<br>26 (20.0%)<br>6 (21.4%) | <br>4<br>6 | 1<br>22<br> | | | 0%<br>15%<br>100% |
| All Cupressales | | 36 | 159 | 33 (20.8%) | 10 | 23 | | | 30% |
| **All Gymnosperms** | | **83** | **990** | **86 (8.7%)** | **27** | **59** | | | **31.4%** |

# Table S4: The Distribution of Ecdysteroid-Positive Species Within Angiosperm Orders; Data from the Ecdybase Literature Survey (ELS)

Orders and Families in the angiosperms are listed according to APGIV (Bot J Linn Soc 181, 1-20, 2016). There are 64 Orders of angiosperms according to this classification. The numbers of genera and species in each family are taken from Christenhusz MJM & Byng JW (Phytotaxa 261(3), 201-217, 2016), which uses the classification of APGIV. The linear sequence of Orders is indicated by letters in square brackets ([A]→[Z]→[AA]→[ZZ]→[AAA]→[LLL]). The linear sequence of Families is indicated by numbers in square brackets ([1]→[416]). The Table is organised primarily according to the evolutionary sequence of the Orders, and secondarily alphabetically according to Family name, but the evolutionary sequence is provided by the Linear Sequence Numbers.

The data on the presence or absence of ecdysteroids derives from the Ecdybase Literature Survey Version 11 (accessed on the 20/08/2021).

| Sequence | Angiosperm Order | Families + [linear sequence number] | No. Genera | No. Species | No. Species Assessed | No. Species Positive | No. Species Negative | No. Species Uncertain | % of All Species Positive | % of Assessed Species Positive |
|---|---|---|---|---|---|---|---|---|---|---|
| [A] | Amborellales<br>[independent clade] | Amborellaceae [1] | 1 | 1 | | | | | | **no data** |
| [B] | Nympheales<br>[Independent clade] | Hydatellaceae [2]<br>Cabombaceae [3]<br>Nymphaeaceae [4] | 1<br>2<br>5 | 12<br>6<br>70 | <br><br>2 | | <br><br>2 | | | |
| | **All Nympheales** | **3** | **8** | **88** | **2 (2.27%)** | **0** | **2** | **0** | **0%** | **0%** |
| [C] | Austrobaileyales<br>[Independent clade] | Austobaileyaceae [5]<br>Trimeniaceae [6]<br>Schisandraceae [7] | 1<br>1<br>3 | 1<br>8<br>85 | | | | | | |
| | **All Austrobaileyales** | **3** | **5** | **94** | | | | | | **no data** |
| [D] | Canellales<br>[Magnolids] | Canellaceae [8]<br>Winteraceae [9] | 5<br>5 | 23<br>65 | | | | | | |
| | **All Canellales** | **2** | **10** | **88** | | | | | | **no data** |
| [E] | Piperales<br>[Mangolids] | Saururaceae [10]<br>Piperaceae [11]<br>Aristolochiaceae [12] | 4<br>5<br>7 | 6<br>3700<br>500 | 1<br>1 | | <br>1 | | | |
| | **All Piperales** | **3** | **16** | **4206** | **1 (0.02%)** | **0** | **1** | **0** | **0%** | **0%** |
| [F] | Magnoliales<br>[Magnolids] | Myristicaceae [13]<br>Magnoliaceae [14]<br>Degeneriaceae [15]<br>Himantandraceae [16]<br>Eupomatiaceae [17] | 21<br>2<br>1<br>1<br>1 | 520<br>294<br>2<br>3<br>2 | 1<br>2 | | 1<br>2 | | | |

| | | | | | | | | | | |
|---|---|---|---|---|---|---|---|---|---|---|
| | | Annonaceae [18] | 105 | 2500 | 2 | | 2 | | | |
| | **All Magnoliales** | **6** | **131** | **3321** | **5 (0.15%)** | **0** | **5** | **0** | **0%** | **0%** |
| [G] | Laurales [Magnolids] | Anthereospermataceae [22] | 6 | 16 | | | | | | |
| | | Calycathaceae [19] | 3 | 10 | | | | | | |
| | | Gomortegaceae [21] | 1 | 1 | | | | | | |
| | | Hernandiaceae [23] | 5 | 58 | | | | | | |
| | | Lauraceae [25] | 45 | 2850 | 1 | | 1 | | | |
| | | Monimiaceae [24] | 24 | 217 | | | | | | |
| | | Siparunaceae [20] | 2 | 75 | | | | | | |
| | **All Laurales** | **7** | **86** | **3227** | **1 (0.03%)** | **0** | **1** | **0** | **0%** | **0%** |
| [H] | Chloranthales [Independent clade] | Chloranthaceae [26] | **4** | **77** | **1 (1.30%)** | **1** | | | **1.30%** | **100%** |
| [I] | Acorales [Monocots] | Acoraceae [27] | 1 | 2 | | | | | | **no data** |
| [J] | Alismatales [Monocots] | Alismataceae [30] | 17 | 115 | 3 | | 3 | | | |
| | | Aponogetonaceae [34] | 1 | 56 | | | | | | |
| | | Araceae [28] | 114 | 3750 | 8 | 2 | 6 | | | |
| | | Butomaceae [31] | 1 | 1 | 1 | 1 | | | | |
| | | Cymodoceaceae [41] | 5 | 17 | | | | | | |
| | | Hydrocharitaceae [32] | 16 | 135 | 3 | 1 | 2 | | | |
| | | Juncaginaceae [35] | 3 | 34 | | | | | | |
| | | Maundiaceae [36] | 1 | 1 | | | | | | |
| | | Posidoniaceae [39] | 1 | 9 | | | | | | |
| | | Potamogetonaceae [38] | 6 | 110 | 8 | 3 | | 5 | | |
| | | Ruppiaceae [40] | 1 | 8 | | | | | | |
| | | Scheuchzeriaceae [33] | 1 | 1 | | | | | | |
| | | Tofieldiaceae [29] | 4 | 28 | | | | | | |
| | | Zosteraceae [37] | 2 | 22 | 1 | | 1 | | | |
| | **All Alismatales** | **14** | **173** | **4287** | **24 (0.56%)** | **7** | **12** | **5** | **0.163%** | **29.2%** |
| [K] | Petrosaviales [Monocots] | Petrosaviaceae [42] | 2 | 4 | | | | | | **no data** |
| [L] | Dioscoreales [Monocots] | Burmanniaceae [44] | 8 | 99 | | | | | | |
| | | Dioscoreaceae [45] | 9 | 715 | 2 | 2 | | | | |
| | | Nartheciaceae [43] | 5 | 38 | | | | | | |
| | **All Dioscoreales** | **3** | **22** | **852** | **2 (0.24%)** | **2** | **0** | **0** | **0.235%** | **100%** |
| [M] | Pandanales [Monocots] | Cyclanthaceae [49] | 12 | 230 | | | | | | |
| | | Pandanaceae [50] | 5 | 982 | | | | | | |
| | | Stemonaceae [48] | 4 | 37 | | | | | | |
| | | Triuridaceae [46] | 9 | 55 | | | | | | |
| | | Velloziaceae [47] | 5 | 306 | | | | | | |
| | **All Pandanales** | **5** | **35** | **1610** | | | | | | **no data** |
| [N] | Liliales | Alstroemeriaceae [55] | 4 | 254 | 1 | 1 | | | | |

| | | | | | | | | | | |
|---|---|---|---|---|---|---|---|---|---|---|
| | [Monocots] | Campynemataceae [51] | 2 | 4 | | | | | | |
| | | Colchicaceae [56] | 15 | 285 | 3 | 1 | 2 | | | |
| | | Corsiaceae [52] | 3 | 27 | | | | | | |
| | | Liliaceae [60] | 15 | 705 | 33 | 1 | 30 | 2 | | |
| | | Melanthiaceae [53] | 17 | 173 | 19 | 18 | 18 | 1 | | |
| | | Petermanniaceae [54] | 1 | 1 | | | | | | |
| | | Philesiaceae [57] | 2 | 2 | | | | | | |
| | | Ripogonaceae [58] | 1 | 6 | | | | | | |
| | | Smilacaceae [59] | 1 | 255 | | | | | | |
| | **All Liliales** | **10** | **61** | **1712** | **56 (3.27%)** | **21** | **33** | **2** | **1.227%** | **37.5%** |
| [P] | Arecales [Monocots] | Arecaceae [76] | 181 | 2600 | 2 | | 2 | | | |
| | | Dasypogonaceae [75] | 4 | 16 | | | | | | |
| | **All Arecales** | **2** | **185** | **2616** | **2 (0.08%)** | **0** | **2** | **0** | **0%** | **0%** |
| [O] | Asparagales [Monocots] | Amaryllidaceae [73] | 75 | 1600 | 20 | 8 | 11 | 1 | | |
| | | Asparagaceae [74] | 114 | 2900 | 35 | 14 | 21 | | | |
| | | Asphodelaceae [72] | 39 | 900 | 2 | | 2 | | | |
| | | Asteliaceae [64] | 3 | 37 | | | | | | |
| | | Blandfordiaceae [63] | 1 | 4 | 2 | 2 | | | | |
| | | Boryaceae [62] | 2 | 12 | | | | | | |
| | | Doryanthaceae [67] | 1 | 2 | | | | | | |
| | | Hypoxidaceae [66] | 4 | 159 | | | | | | |
| | | Iridaceae [70] | 66 | 2244 | 9 | 4 | 5 | | | |
| | | Ixioliriaceae [68] | 1 | 4 | | | | | | |
| | | Lanariaceae [65] | 1 | 1 | | | | | | |
| | | Orchidaceae [61] | 736 | 28000 | 9 | 1 | 8 | | | |
| | | Tecophilaeaceae [69] | 9 | 27 | | | | | | |
| | | Xeronemataceae [71] | 1 | 2 | | | | | | |
| | **All Asparagales** | **14** | **1053** | **35892** | **77 (0.22%)** | **29** | **47** | **1** | **0.081%** | **37.7%** |
| [Q] | Commelinales [Monocots] | Commelinaceae [78] | 41 | 731 | 66 | 55 | 7 | 4 | | |
| | | Haemodoraceae [81] | 14 | 102 | | | | | | |
| | | Hanguanaceae [77] | 1 | 12 | | | | | | |
| | | Philydraceae [79] | 3 | 6 | | | | | | |
| | | Pontederiaceae [80] | 6 | 34 | | | | | | |
| | **All Commelinales** | **5** | **65** | **885** | **66 (7.46%)** | **55** | **7** | **4** | **6.22%** | **83.3%** |
| [R] | Zingiberales [Monocots] | Cannaceae [86] | 1 | 10 | | | | | | |
| | | Costaceae [88] | 7 | 143 | | | | | | |
| | | Heliconiaceae [84] | 1 | 194 | | | | | | |
| | | Lowiaceae [83] | 1 | 18 | | | | | | |
| | | Marantaceae [87] | 29 | 570 | | | | | | |
| | | Musaceae [85] | 3 | 91 | 1 | | 1 | | | |
| | | Strelitziaceae [82] | 3 | 7 | | | | | | |
| | | Zingiberaceae [89] | 50 | 1600 | 3 | | 3 | | | |
| | **All Zingiberales** | **8** | **95** | **2633** | **4 (0.15%)** | **0** | **4** | **0** | **0%** | **0%** |

| | | | | | | | | | | |
|---|---|---|---|---|---|---|---|---|---|---|
| [S] | Poales | Bromeliaceae [91] | 51 | 3475 | 3 | | 3 | | | |
| | [Monocots] | Cyperaceae [98] | 9 | 5500 | 37 | | 35 | 2 | | |
| | | Ecdeiocoleaceae [102] | 2 | 3 | | | | | | |
| | | Eriocaulaceae [94] | 7 | 1207 | | | | | | |
| | | Flagellariaceae [100] | 1 | 4 | | | | | | |
| | | Joinvilleaceae [101] | 1 | 4 | | | | | | |
| | | Juncaceae [97] | 8 | 464 | 4 | | 4 | | | |
| | | Mayacaceae [95] | 1 | 6 | | | | | | |
| | | Poaceae [103] | 780 | 12000 | 85 | 8 | 74 | 2 | | |
| | | Rapateaceae [92] | 16 | 94 | | | | | | |
| | | Restionaceae [99] | 51 | 572 | | | | | | |
| | | Thurniaceae [96] | 2 | 4 | | | | | | |
| | | Typhaceae [90] | 2 | 51 | 3 | | 3 | | | |
| | | Xyridaceae [93] | 5 | 399 | | | | | | |
| | **All Poales** | **15** | **936** | **23783** | **132 (0.56%)** | **9** | **119** | **4** | **0.038%** | **6.8%** |
| [T] | Ceratophyllales | Ceratophyllaceae [104] | **1** | **4** | **1 (25.0%)** | **1** | | | **25.0%** | **100%** |
| | [Sister of Eudicots] | | | | | | | | | |
| [U] | Ranunculales | Berberidaceae [110] | 14 | 700 | | | | | | |
| | [Eudicots] | Circaeasteraceae [107] | 2 | 2 | | | | | | |
| | | Eupteleaceae [105] | 1 | 2 | | | | | | |
| | | Lardizabalaceae [108] | 7 | 40 | 2 | 1 | 1 | | | |
| | | Menispermaceae [109] | 68 | 440 | 25 | 16 | 9 | | | |
| | | Papaveraceae [106] | 42 | 775 | 7 | 1 | 6 | | | |
| | | Ranunculaceae [111] | 43 | 2346 | 184 | 51 | 126 | 7 | | |
| | **All Ranunculales** | **7** | **177** | **4305** | **218 (5.06%)** | **69** | **142** | **7** | **1.603%** | **53.9%** |
| [V] | Proteales | Nelumbonaceae [113] | 1 | 3 | 1 | | 1 | | | |
| | [Eudicots] | Platanaceae [114] | 1 | 8 | 1 | | 1 | | | |
| | | Proteaceae [115] | 83 | 1660 | 1 | | 1 | | | |
| | | Sabiaceae [112] | 3 | 66 | | | | | | |
| | **All Proteales** | **4** | **88** | **1737** | **3 (0.17%)** | **0** | **3** | **0** | **0%** | **0%** |
| [W] | Trochodendrales | Trochodendraceae [116] | 2 | 2 | | | | | | **no data** |
| | [Eudicots] | | | | | | | | | |
| [X] | Buxales | Buxaceae [117] | 6 | 123 | | | | | | **no data** |
| | [Eudicots] | | | | | | | | | |
| [Y] | Gunnerales | Gunneraceae [119] | 1 | 63 | | | | | | |
| | [Core Eudicots] | Myrothamnaceae [118] | 1 | 2 | | | | | | |
| | **All Gunnerales** | **2** | **2** | **65** | | | | | | **no data** |
| [Z] | Dilleniales | Dilleniaceae [120] | 11 | 430 | | | | | | **no data** |
| | [Core Eudicots] | | | | | | | | | |
| [AA] | Saxifragales | Altingiaceae [123] | 1 | 15 | 1 | | 1 | | | |
| | [Eudicots – | Aphanopetalaceae [131] | 1 | 2 | 1 | 1 | | | | |
| | Superrosids] | Cercidiphyllaceae [125] | 1 | 2 | | | | | | |

| | | | | | | | | | | |
|---|---|---|---|---|---|---|---|---|---|---|
| | | Crassulaceae [130] | 35 | 1400 | 9 | 2 | 7 | | | |
| | | Cynomoriaceae [135] | 1 | 2 | | | | | | |
| | | Daphniphyllaceae [126] | 1 | 30 | | | | | | |
| | | Grossulariaceae [128] | 1 | 150 | 4 | | 4 | | | |
| | | Haloragaceae [134] | 19 | 145 | 1 | | 1 | | | |
| | | Hamamelidaceae [124] | 26 | 86 | | | | | | |
| | | Iteaceae [127] | 2 | 18 | | | | | | |
| | | Paeoniaceae [122] | 1 | 33 | 2 | | 2 | | | |
| | | Penthoraceae [133] | 1 | 2 | | | | | | |
| | | Peridiscaceae [121] | 4 | 12 | | | | | | |
| | | Saxifragaceae [129] | 33 | 640 | 8 | 1 | 7 | | | |
| | | Tetracarpaeaceae [132] | 1 | 1 | | | | | | |
| | **All Saxifragales** | **15** | **128** | **2538** | **26 (1.02%)** | **4** | **22** | **0** | **0.158%** | **15.4%** |
| [BB] | Vitales<br>[Eudicots – Rosids] | Vitaceae [136] | **14** | **910** | **3 (0.33%)** | **0** | **3** | **0** | **0%** | **0%** |
| [CC] | Zygophyllales<br>[Eudicots – Rosids] | Krameriaceae [137] | 1 | 18 | | | | | | |
| | | Zygophyllaceae [138] | 22 | 285 | | | | | | |
| | **All Zygophyllales** | **2** | **23** | **303** | | | | | | **no data** |
| [DD] | Fabales<br>[Eudicots – Rosids] | Fabaceae [140] | 751 | 19500 | 40 | 8 | 32 | | | |
| | | Polygalaceae [142] | 21 | 900 | 1 | | 1 | | | |
| | | Quillajaceae [139] | 1 | 3 | | | | | | |
| | | Surianaceae [141] | 5 | 8 | | | | | | |
| | **All Fabales** | **4** | **778** | **20411** | **41 (0.20%)** | **8** | **33** | **0** | **0.039%** | **19.5%** |
| [EE] | Rosales<br>[Eudicots – Rosids] | Barbeyaceae [144] | 1 | 1 | | | | | | |
| | | Cannabaceae [149] | 8 | 100 | 2 | 1 | 1 | | | |
| | | Dirachmaceae [145] | 1 | 2 | | | | | | |
| | | Elaeagnaceae [146] | 3 | 60 | | | | | | |
| | | Moraceae [150] | 38 | 1180 | 7 | 2 | 4 | 1 | | |
| | | Rhamnaceae [147] | 55 | 950 | 5 | 1 | 4 | | | |
| | | Rosaceae [143] | 91 | 2950 | 45 | 4 | 41 | | | |
| | | Ulmaceae [148] | 7 | 45 | 1 | | 1 | | | |
| | | Urticaceae [151] | 53 | 2625 | 5 | | 5 | | | |
| | **All Rosales** | **9** | **257** | **7913** | **65 (0.82%)** | **8** | **56** | **1** | **0.101%** | **12.3%** |
| [FF] | Fagales<br>[Eudicots – Rosids] | Betulaceae [158] | 6 | 167 | 8 | 4 | 4 | | | |
| | | Casuarinaceae [156] | 4 | 91 | 1 | | 1 | | | |
| | | Fagaceae [153] | 8 | 927 | 4 | | 4 | | | |
| | | Juglandaceae [155] | 9 | 50 | 3 | | 3 | | | |
| | | Myricaceae [154] | 3 | 57 | | | | | | |
| | | Nothofagaceae [152] | 1 | 48 | | | | | | |
| | | Ticodendraceae [157] | 1 | 1 | | | | | | |
| | **All Fagales** | **7** | **32** | **1341** | **16 (1.19%)** | **4** | **12** | **0** | **0.298%** | **25.0%** |
| [GG] | Cucurbitales | Anisophyllaceae [160] | 4 | 71 | | | | | | |

| | | | | | | | | | | |
|---|---|---|---|---|---|---|---|---|---|---|
| | [Eudicots – Rosids] | Apodanthaceae [159] | 2 | 10 | | | | | | |
| | | Begoniaceae [166] | 2 | 1825 | | | | | | |
| | | Coriariaceae [162] | 1 | 14 | 1 | 1 | | | | |
| | | Corynocarpaceae [161] | 1 | 5 | | | | | | |
| | | Cucurbitaceae [163] | 95 | 965 | 9 | 1 | 7 | 1 | | |
| | | Datiscaceae [165] | 1 | 3 | | | | | | |
| | | Tetramelaceae [164] | 2 | 2 | | | | | | |
| | **All Cucurbitales** | **8** | **110** | **2895** | **10 (0.35%)** | **2** | **7** | **1** | **0.069%** | **20.0%** |
| [HH] | Celeastrales [Eudicots – Rosids] | Celastraceae [168] | 96 | 1350 | 6 | 1 | 5 | | | |
| | | Lepidobotryaceae [167] | 2 | 2 | | | | | | |
| | **All Celastrales** | **2** | **98** | **1352** | **6 (0.44%)** | **1** | **5** | **0** | **0.074%** | **16.6%** |
| [II] | Oxalidales [Eudicots – Rosids] | Brunelliaceae [175] | 1 | 60 | | | | | | |
| | | Cephalotaceae [174] | 1 | 1 | | | | | | |
| | | Connaraceae [170] | 12 | 180 | | | | | | |
| | | Cunoniaceae [172] | 27 | 330 | 1 | 1 | | | | |
| | | Elaeocarpaceae [173] | 12 | 615 | | | | | | |
| | | Huaceae [169] | 2 | 4 | | | | | | |
| | | Oxalidaceae [171] | 5 | 570 | 3 | 1 | 1 | 1 | | |
| | **All Oxalidales** | **7** | **60** | **1760** | **4 (0.23%)** | **2** | **1** | **1** | **0.114%** | **50.0%** |
| [JJ] | Malpighiales [Eudicots – Rosids] | Achariaceae [199] | 32 | 155 | | | | | | |
| | | Balanopacaceae [193] | 1 | 9 | | | | | | |
| | | Bonnetiaceae [182] | 3 | 35 | | | | | | |
| | | Calophyllaceae [184] | 14 | 475 | | | | | | |
| | | Caryocaraceae [187] | 2 | 26 | | | | | | |
| | | Centroplacaceae [190] | 2 | 6 | | | | | | |
| | | Chrysobalanaceae [197] | 18 | 533 | | | | | | |
| | | Clusiaceae [183] | 13 | 750 | 1 | | 1 | | | |
| | | Ctenolophonaceae [178] | 1 | 2 | | | | | | |
| | | Dichapetalaceae [195] | 3 | 170 | | | | | | |
| | | Elatinaceae [191] | 2 | 35 | | | | | | |
| | | Erythroxylaceae [180] | 4 | 242 | | | | | | |
| | | Euphorbiaceae [207] | 209 | 6252 | 12 | 2 | 10 | | | |
| | | Euphroniaceae [196] | 1 | 3 | | | | | | |
| | | Goupiaceae [201] | 1 | 2 | | | | | | |
| | | Humiriaceae [198] | 5 | 56 | | | | | | |
| | | Hypericaceae [186] | 6 | 590 | 4 | 1 | 3 | | | |
| | | Irvingiaceae [177] | 3 | 13 | | | | | | |
| | | Ixonanthaceae [209] | 3 | 17 | | | | | | |
| | | Lacistemataceae [203] | 2 | 14 | | | | | | |
| | | Linaceae [208] | 10 | 255 | 1 | | 1 | | | |
| | | Lophopyxidaceae [188] | 1 | 1 | | | | | | |
| | | Malphigiaceae [192] | 73 | 1315 | 1 | 1 | | | | |
| | | Ochnaceae [181] | 32 | 550 | 1 | 1 | | | | |

| | | | | | | | | | | |
|---|---|---|---|---|---|---|---|---|---|---|
| | | Pandaceae [176] | 3 | 17 | | | | | | |
| | | Passifloraceae [202] | 29 | 980 | 2 | | 2 | | | |
| | | Peraceae [205] | 5 | 127 | | | | | | |
| | | Phyllanthaceae [211] | 57 | 2050 | 4 | 1 | 3 | | | |
| | | Picrodendraceae [210] | 25 | 96 | | | | | | |
| | | Podostemaceae [185] | 46 | 300 | | | | | | |
| | | Putranjivaceae [189] | 2 | 216 | | | | | | |
| | | Rafflesiaceae [206] | 3 | 25 | | | | | | |
| | | Rhizophoraceae [179] | 15 | 147 | | | | | | |
| | | Salicaceae [204] | 56 | 1220 | 9 | | 9 | | | |
| | | Trigoniaceae [194] | 5 | 28 | | | | | | |
| | | Violaceae [200] | 31 | 980 | 3 | | 3 | | | |
| | **All Malphigiales** | **36** | **717** | **17692** | **38 (0.22%)** | **6** | **32** | **0** | **0.034%** | **15.8%** |
| [KK] | Geraniales [Eudicots – Rosids] | Francoaceae [213] | 8 | 37 | | | | | | |
| | | Geraniaceae [212] | 5 | 830 | 7 | 1 | 6 | | | |
| | **All Gerianales** | **2** | **13** | **867** | **7 (0.81%)** | **1** | **6** | **0** | **0.115%** | **14.3%** |
| [LL] | Myrtales [Eudicots – Rosids] | Alzateaceae [221] | 1 | 1 | | | | | | |
| | | Combretaceae [214] | 10 | 530 | 1 | | 1 | | | |
| | | Crypteroniaceae [220] | 3 | 13 | | | | | | |
| | | Lythraceae [215] | 27 | 620 | 1 | | 1 | | | |
| | | Melastomataceae [219] | 165 | 5115 | | | | | | |
| | | Myrtaceae [218] | 132 | 5950 | 2 | | 2 | | | |
| | | Onagraceae [216] | 22 | 656 | 8 | 1 | 7 | | | |
| | | Penaeaceae [222] | 9 | 32 | | | | | | |
| | | Vochysiaceae [217] | 7 | 217 | | | | | | |
| | **All Myrtales** | **9** | **376** | **13134** | **12 (0.09%)** | **1** | **11** | **0** | **0.008%** | **8.3%** |
| [MM] | Crossosomatales [Eudicots – Rosids] | Asphloaceae [223] | 1 | 1 | | | | | | |
| | | Crossosomataceae [229] | 4 | 10 | | | | | | |
| | | Geissolomataceae [224] | 1 | 1 | | | | | | |
| | | Guamatelaceae [227] | 1 | 1 | | | | | | |
| | | Stachyuraceae [228] | 1 | 8 | 2 | 2 | | | | |
| | | Staphyleaceae [226] | 2 | 45 | | | | | | |
| | | Strasburgeriaceae [225] | 2 | 2 | | | | | | |
| | **All Crossosomatales** | **7** | **12** | **68** | **2 (2.94%)** | **2** | **0** | **0** | **2.941%** | **100%** |
| [NN] | Picramniales [Eudicots –Rosids] | Picramniaceae [230] | 3 | 49 | | | | | | **no data** |
| [OO] | **H**uerteales [Eudicots – Rosids] | Dipentodontaceae [234] | 2 | 20 | | | | | | |
| | | Gerrardinaceae [231] | 1 | 2 | | | | | | |
| | | Petenaeceae [232] | 1 | 1 | | | | | | |
| | | Tapisciaceae [233] | 2 | 6 | | | | | | |
| | **All Huerteales** | **4** | **6** | **29** | | | | | | **no data** |

| | | | | | | | | | | |
|---|---|---|---|---|---|---|---|---|---|---|
| [PP] | Sapindales [Eudicots – Rosids] | Anacardiaceae [239] | 83 | 860 | 5 | 2 | 3 | | | |
| | | Burseraceae [238] | 19 | 615 | | | | | | |
| | | Biebersteiniaceae [235] | 1 | 4 | | | | | | |
| | | Kirkiaceae [237] | 1 | 6 | | | | | | |
| | | Meliaceae [243] | 53 | 600 | 2 | | 2 | | | |
| | | Nitrariaceae [236] | 3 | 19 | | | | | | |
| | | Rutaceae [241] | 148 | 2070 | 9 | 4 | 4 | 1 | | |
| | | Sapindaceae [240] | 142 | 1860 | 6 | | 6 | | | |
| | | Simaroubaceae [242] | 22 | 108 | 2 | 1 | 1 | | | |
| | **All Sapindales** | **9** | **472** | **6142** | **24 (0.39%)** | **7** | **16** | **1** | **0.114%** | **29.2%** |
| [QQ] | Malvales [Eudicots –Rosids] | Bixaceae [250] | 4 | 23 | | | | | | |
| | | Cistaceae [251] | 9 | 170 | 6 | 4 | 2 | | | |
| | | Cytinaceae [244] | 2 | 10 | | | | | | |
| | | Dipterocarpaceae [253] | 16 | 695 | | | | | | |
| | | Malvaceae [247] | 244 | 4225 | 36 | 17 | 17 | 2 | | |
| | | Muntingiaceae [245] | 3 | 3 | | | | | | |
| | | Neuradaceae [246] | 3 | 10 | | | | | | |
| | | Sarcolaenaceae [252] | 10 | 71 | | | | | | |
| | | Sphaerosepalaceae [248] | 2 | 18 | | | | | | |
| | | Thymelaeaceae [249] | 46 | 913 | 1 | | 1 | | | |
| | **All Malvales** | **10** | **339** | **6138** | **43 (0.70%)** | **21** | **20** | **2** | **0.342%** | **48.8%** |
| [RR] | Brassicales [Eudicots – Rosids] | Akaniaceae [254] | 2 | 2 | | | | | | |
| | | Bataceae [261] | 1 | 2 | | | | | | |
| | | Brassicaceae [270] | 328 | 3628 | 28 | 4 | 24 | | | |
| | | Capparaceae [268] | 30 | 324 | 2 | 2 | | | | |
| | | Caricaceae [257] | 6 | 35 | 1 | | 1 | | | |
| | | Cleomaceae [269] | 1 | 346 | 2 | | 2 | | | |
| | | Emblingiaceae [263] | 1 | 1 | | | | | | |
| | | Gyrostemonaceae [266] | 4 | 20 | | | | | | |
| | | Koeberliniaceae [260] | 1 | 2 | | | | | | |
| | | Limnanthaceae [258] | 2 | 8 | 6 | 6 | | | | |
| | | Moringaceae [256] | 1 | 13 | 1 | | 1 | | | |
| | | Pentadiplandraceae [265] | 1 | 1 | | | | | | |
| | | Resedaceae [267] | 12 | 107 | | | | | | |
| | | Salvadoraceae [262] | 3 | 11 | | | | | | |
| | | Setchellanthaceae [259] | 1 | 1 | | | | | | |
| | | Tovariaceae [264] | 1 | 2 | | | | | | |
| | | Tropaeolaceae [255] | 1 | 94 | 3 | | 3 | | | |
| | **All Brassicales** | **17** | **396** | **4596** | **43 (0.94%)** | **12** | **31** | **0** | **0.261%** | **27.9%** |
| [SS] | Berberidopsidales [Eudicots – Superasterids] | Aextoxicaceae [271] | 1 | 1 | | | | | | |
| | | Berberidopsidaceae [272] | 2 | 3 | | | | | | |
| | **All** | **2** | **3** | **4** | | | | | | **no data** |

| | | | | | | | | | | |
|---|---|---|---|---|---|---|---|---|---|---|
| | **Berberidopsidales** | | | | | | | | | |
| [TT] | Santalales [Eudicots – Superasterids] | Balanophoraceae [275] | 17 | 44 | | | | | | |
| | | Loranthaceae [279] | 76 | 1050 | | | | | | |
| | | Misodendraceae [277] | 1 | 8 | | | | | | |
| | | Olacaceae [273] | 29 | 180 | | | | | | |
| | | Opiliaceae [274] | 11 | 33 | | | | | | |
| | | Santalaceae [276] | 43 | 1000 | | | | | | |
| | | Schoepfiaceae [278] | 3 | 58 | | | | | | |
| | **All Santalales** | **7** | **180** | **2373** | | | | | | **no data** |
| [UU] | Caryophyllales [Eudicots – Superasterids] | Achatocarpaceae [296] | 2 | 11 | | | | | | |
| | | Aizoaceae [304] | 121 | 1900 | 6 | 5 | 1 | | | |
| | | Amaranthaceae [297] | 165 | 2040 | 273 | 116 | 119 | 38 | | |
| | | Anacampserotaceae [316] | 3 | 36 | | | | | | |
| | | Ancistrocladaceae [288] | 1 | 21 | | | | | | |
| | | Asteropeiaceae [292] | 1 | 8 | | | | | | |
| | | Barbeuiaceae [302] | 1 | 1 | | | | | | |
| | | Basellaceae [312] | 4 | 19 | | | | | | |
| | | Cactaceae [317] | 127 | 1750 | 5 | 1 | 3 | 1 | | |
| | | Caryophyllaceae [295] | 81 | 2625 | 324 | 180 | 115 | 29 | | |
| | | Didiereaceae [311] | 7 | 22 | | | | | | |
| | | Dioncophyllaceae [287] | 3 | 3 | | | | | | |
| | | Droseraceae [284] | 3 | 180 | 1 | | 1 | | | |
| | | Drosphyllaceae [286] | 1 | 1 | | | | | | |
| | | Frankeniaceae [280] | 1 | 90 | | | | | | |
| | | Gisekiaceae [303] | 1 | 8 | | | | | | |
| | | Halophytaceae [313] | 1 | 1 | | | | | | |
| | | Kewaceae [301] | 1 | 8 | | | | | | |
| | | Limeaceae [299] | 1 | 21 | | | | | | |
| | | Lophiocarpaceae [300] | 2 | 6 | | | | | | |
| | | Macarthuriaceae [293] | 1 | 10 | | | | | | |
| | | Microteaceae [294] | 1 | 9 | | | | | | |
| | | Molluginaceae [309] | 9 | 80 | | | | | | |
| | | Montiaceae [310] | 14 | 230 | 1 | | 1 | | | |
| | | Nepenthaceae [285] | 1 | 150 | | | | | | |
| | | Nyctaginaceae [308] | 31 | 400 | 3 | 1 | 1 | 1 | | |
| | | Petiveriaceae [306] | 9 | 20 | 2 | | 2 | | | |
| | | Physenaceae [291] | 1 | 2 | | | | | | |
| | | Phytolaccaceae [305] | 5 | 33 | 3 | | 3 | | | |
| | | Plumbaginaceae [282] | 30 | 725 | 18 | 7 | 11 | | | |
| | | Polygonaceae [283] | 48 | 1200 | 15 | | 13 | 2 | | |
| | | Portulacaceae [315] | 1 | 115 | 3 | | 2 | 1 | | |
| | | Rhabdodendraceae [289] | 1 | 3 | | | | | | |

| | | | | | | | | | | |
|---|---|---|---|---|---|---|---|---|---|---|
| | | Sarcobataceae [307] | 1 | 2 | | | | | | |
| | | Simmondsiaceae [290] | 1 | 1 | | | | | | |
| | | Stegnospermataceae [298] | 1 | 4 | | | | | | |
| | | Talinaceae [314] | 2 | 28 | | | | | | |
| | | Tamaricaceae [281] | 4 | 78 | | | | | | |
| | **All Caryophyllales** | **38** | **688** | **11841** | **654 (5.52%)** | **310** | **272** | **72** | **2.618%** | **47.4%** |
| [VV] | Cornales [Eudicots – Asterids] | Cornaceae [324] | 2 | 85 | 7 | | 7 | | | |
| | | Curtisiaceae [322] | 1 | 1 | | | | | | |
| | | Grubbiaceae [323] | 1 | 3 | | | | | | |
| | | Hydrangeaceae [320] | 9 | 223 | 3 | 1 | 2 | | | |
| | | Hydrostachyaceae [319] | 1 | 22 | | | | | | |
| | | Loasaceae [321] | 20 | 308 | 1 | | 1 | | | |
| | | Nyssaceae [318] | 5 | 37 | | | | | | |
| | **All Cornales** | **7** | **39** | **679** | **11 (1.62%)** | **1** | **10** | **0** | **0.147%** | **9.1%** |
| [WW] | Ericales [Eudicots – Asterids] | Actinidiaceae [342] | 3 | 360 | 3 | | 3 | | | |
| | | Balsaminaceae [325] | 2 | 1000 | | | | | | |
| | | Clethraceae [343] | 2 | 75 | | | | | | |
| | | Cyrillaceae [344] | 2 | 2 | | | | | | |
| | | Diapensiaceae [338] | 5 | 12 | 1 | | 1 | | | |
| | | Ebenaceae [334] | 4 | 800 | 2 | | 1 | 1 | | |
| | | Ericaceae [345] | 124 | 4250 | 18 | | 18 | | | |
| | | Fouquieriaceae [328] | 1 | 11 | | | | | | |
| | | Lecythidaceae [330] | 25 | 355 | | | | | | |
| | | Marcgraviaceae [326] | 7 | 120 | | | | | | |
| | | Mitrastemonaceae [346] | 1 | 2 | | | | | | |
| | | Pentaphylaceae [332] | 12 | 330 | | | | | | |
| | | Polemoniaceae [329] | 26 | 350 | 5 | 3 | 2 | | | |
| | | Primulaceae [335] | 53 | 2790 | 23 | 7 | 16 | | | |
| | | Roridulaceae [341] | 1 | 2 | | | | | | |
| | | Sapotaceae [333] | 54 | 1273 | 1 | | 1 | | | |
| | | Sarraceniaceae [340] | 3 | 34 | | | | | | |
| | | Sladeniaceae [331] | 2 | 3 | | | | | | |
| | | Styracaceae [339] | 11 | 160 | | | | | | |
| | | Symplocaceae [337] | 2 | 260 | | | | | | |
| | | Tetrameristaceae [327] | 3 | 5 | | | | | | |
| | | Theaceae [336] | 9 | 240 | | | | | | |
| | **All Ericales** | **22** | **352** | **12435** | **53 (0.43%)** | **10** | **42** | **1** | **0.080%** | **18.9%** |
| [XX] | Icacinales [Eudicots – Asterids] | Icacinaceae [348] | 25 | 165 | | | | | | |
| | | Oncothecaceae [347] | 1 | 2 | | | | | | |
| | **All Icacinalaes** | **2** | **26** | **167** | | | | | | **no data** |
| [YY] | Metteniusales [Eudicots – Asterids] | Metteniusaceae [349] | 11 | 50 | | | | | | **no data** |

| | | | | | | | | | | |
|---|---|---|---|---|---|---|---|---|---|---|
| [ZZ] | Garryales | Eucommiaceae [350] | 1 | 1 | | | | | | |
| | [Eudicots – Asterids] | Garryaceae [351] | 2 | 25 | 4 | 2 | 2 | | | |
| | **All Garryales** | **2** | **3** | **26** | **4 (15.4%)** | **2** | **2** | **0** | **7.69%** | **50.0%** |
| [AAA] | Gentianales | Apocynaceae [356] | 366 | 5100 | 8 | 4 | 4 | | | |
| | [Eudicots – Asterids] | Gelsemiaceae [355] | 3 | 11 | | | | | | |
| | | Gentianaceae [353] | 102 | 1735 | 8 | 1 | 7 | | | |
| | | Loganiaceae [354] | 15 | 390 | | | | | | |
| | | Rubiaceae [352] | 590 | 13620 | 6 | | 6 | | | |
| | **All Gentianales** | **5** | **1076** | **20856** | **22 (0.11%)** | **5** | **17** | **0** | **0.024%** | **22.7%** |
| [BBB] | Boraginales<br>[Eudicots – Asterids] | Boraginaceae [357] | **135** | **2535** | **15 (0.59%)** | **6** | **8** | **1** | **0.237%** | **40.0%** |
| [CCC] | Vahliales<br>[Eudicots – Asterids] | Vahliaceae [358] | 1 | 8 | | | | | | **no data** |
| [DDD] | Solanales | Convolvulaceae [359] | 53 | 1660 | 11 | 4 | 7 | | | |
| | [Eudicots – Asterids] | Hydroleaceae [363] | 1 | 12 | | | | | | |
| | | Montiniaceae [361] | 3 | 5 | | | | | | |
| | | Solanaceae [360] | 100 | 2600 | 132 | 20 | 85 | 27 | | |
| | | Sphenocleaceae [362] | 1 | 2 | | | | | | |
| | **All Solanales** | **5** | **158** | **4279** | **143 (3.34%)** | **24** | **92** | **27** | **0.561%** | **16.8%** |
| [EEE] | Lamiales | Acanthaceae [377] | 210 | 4000 | 7 | 4 | 3 | | | |
| | [Eudicots – Asterids] | Bignoniaceae [378] | 82 | 870 | 1 | 1 | | | | |
| | | Byblidaceae [374] | 1 | 7 | | | | | | |
| | | Calceolariaceae [368] | 2 | 271 | | | | | | |
| | | Carlemanniaceae [365] | 2 | 5 | | | | | | |
| | | Gesneriaceae [369] | 152 | 3540 | | | | | | |
| | | Lamiaceae [383] | 241 | 7530 | 111 | 69 | 39 | 3 | | |
| | | Lentibulariaceae [379] | 3 | 316 | 1 | | 1 | | | |
| | | Linderniaceae [373] | 23 | 220 | | | | | | |
| | | Martyniaceae [375] | 5 | 16 | 1 | 1 | | | | |
| | | Mazaceae [384] | 3 | 33 | | | | | | |
| | | Oleaceae [366] | 26 | 790 | 11 | 5 | 6 | | | |
| | | Orabanchaceae [387] | 98 | 1960 | 5 | | 5 | | | |
| | | Paulowniaceae [386] | 3 | 8 | 3 | | 3 | | | |
| | | Pedaliaceae [376] | 13 | 75 | 1 | | 1 | | | |
| | | Phyrmaceae [385] | 13 | 136 | 1 | 1 | | | | |
| | | Plantaginaceae [370] | 94 | 1900 | 29 | 13 | 16 | | | |
| | | Plocospermataceae [364] | 1 | 1 | | | | | | |
| | | Schlegeliaceae [380] | 4 | 37 | | | | | | |
| | | Scrophulariaceae [371] | 62 | 1830 | 19 | 5 | 14 | | | |
| | | Stilbaceae [372] | 8 | 40 | | | | | | |
| | | Tetrachondraceae [367] | 2 | 3 | | | | | | |
| | | Thomandersiaceae [381] | 1 | 6 | | | | | | |
| | | Verbenaceae [382] | 32 | 1000 | 1 | | 1 | | | |

| | | | | | | | | | | |
|---|---|---|---|---|---|---|---|---|---|---|
| | **All Lamiales** | **24** | **1080** | **25494** | **191 (0.75%)** | **99** | **89** | **3** | **0.388%** | **51.8%** |
| [FFF] | Aquifoliales [Eudicots – Asterids] | Aquifoliaceae [392] | 1 | 500 | 7 | 4 | 3 | | | |
| | | Cardiopteridaceae [389] | 5 | 43 | | | | | | |
| | | Helwingiaceae [391] | 1 | 4 | | | | | | |
| | | Phyllonomaceae [390] | 1 | 4 | | | | | | |
| | | Stemonuraceae [388] | 12 | 90 | | | | | | |
| | **All Aquifoliales** | **5** | **20** | **641** | **7 (1.09%)** | **4** | **3** | **0** | **0.624%** | **57.1%** |
| [GGG] | Asterales [Eudicots – Asterids] | Alseuosmiaceae [397] | 5 | 13 | | | | | | |
| | | Argophyllaceae [399] | 2 | 21 | | | | | | |
| | | Asteraceae [403] | 1623 | 24700 | 189 | 76 | 100 | 13 | | |
| | | Calyceraceae [402] | 4 | 60 | | | | | | |
| | | Campanulaceae [394] | 81 | 2800 | 9 | 4 | 5 | | | |
| | | Goodeniaceae [401] | 12 | 440 | | | | | | |
| | | Menyanthaceae [400] | 6 | 60 | 1 | | 1 | | | |
| | | Pentaphragmataceae [395] | 1 | 30 | | | | | | |
| | | Phellinaceae [398] | 1 | 12 | | | | | | |
| | | Rousseaceae [393] | 4 | 6 | | | | | | |
| | | Stylidiaceae [396] | 6 | 245 | | | | | | |
| | **All Asterales** | **11** | **1745** | **28387** | **199 (0.70%)** | **80** | **106** | **13** | **0.282%** | **40.2%** |
| [HHH] | Escallioniales [Eudicots – Asterids] | Escalloniaceae [404] | 7 | 103 | | | | | | **no data** |
| [III] | Bruniales [Eudicost – Asterids] | Bruniaceae [406] | 6 | 81 | | | | | | |
| | | Columelliaceae [405] | 2 | 8 | | | | | | |
| | **All Bruniales** | **2** | **8** | **89** | | | | | | **no data** |
| [JJJ] | Paracryphiales [Eudicots – Asterids] | Paracryphiaceae [407] | 3 | 36 | | | | | | **no data** |
| [KKK] | Dipsacales [Eudicots – Asterids] | Adoxaceae [408] | 5 | 225 | 3 | 1 | 1 | 1 | | |
| | | Caprifoliaceae [409] | 28 | 825 | 10 | | 10 | | | |
| | **All Dipsacales** | **2** | **33** | **1050** | **13 (1.24%)** | **1** | **11** | **1** | **0.095%** | **7.7%** |
| [LLL] | Apiales [Eudicots – Asterids] | Apiaceae [416] | 442 | 3575 | 29 | 3 | 26 | | | |
| | | Araliaceae [414] | 43 | 1650 | 5 | | 5 | | | |
| | | Griseliniaceae [412] | 1 | 7 | | | | | | |
| | | Myodocarpaceae [415] | 2 | 15 | | | | | | |
| | | Pennantiaceae [410] | 1 | 4 | | | | | | |
| | | Pittosporaceae [413] | 7 | 245 | 1 | 1 | | | | |
| | | Torricelliaceae [411] | 3 | 10 | | | | | | |
| | **All Apiales** | **7** | **499** | **5506** | **35 (0.64%)** | **4** | **31** | **0** | **0.073%** | **11.4%** |
| | **All Angiosperms** | **416** | **13164** | **295383** | **2282 (0.77%)** | **819** | **1316** | **146** | **0.277%** | **35.9%** |

# Table S5: The Distribution of Ecdysteroid-Positive Species Within Angiosperm Orders; Data from the Exeter Survey (ES)

Orders and Families in the angiosperms are listed according to APGIV (Bot J Linn Soc 181, 1-20, 2016). There are 64 Orders of angiosperms according to this classification. The numbers of genera and species in each family are taken from Christenhusz MJM & Byng JW (Phytotaxa 261(3), 201-217, 2016), which uses the classification of APGIV. The linear sequence of Orders is indicated by letters in square brackets ([A]→[Z]→[AA]→[ZZ]→[AAA]→[LLL]). The linear sequence of Families is indicated by numbers in square brackets ([1]→[416]). The Table is organised primarily according to the evolutionary sequence of the Orders, and secondarily alphabetically according to Family name, but the evolutionary sequence is provided by the Linear Sequence Numbers.

Data on the presence/absence of ecdysteroids derives from the Exeter Survey of plants for ecdysteroid agonists and antagonists (1995-2002; Version 1).

| Sequence | Angiosperm Order | Families + [linear sequence number] | No. Genera | No. Species | No. Species Assessed | No. Species Positive | No. Species Negative | No. Species Uncertain | % of All Species Positive | % of Assessed Species Positive |
|---|---|---|---|---|---|---|---|---|---|---|
| [A] | Amborellales [independent clade] | Amborellaceae [1] | 1 | 1 | | | | | | **no data** |
| [B] | Nympheales [Independent clade] | Cabombaceae [3]<br>Hydatellaceae [2]<br>Nymphaeaceae [4] | 2<br>1<br>5 | 6<br>12<br>70 | <br><br>3 | | <br><br>3 | | | |
| | **All Nympheales** | **3** | **8** | **88** | **3 (3.41%)** | | **3** | | **0%** | **0%** |
| [C] | Austrobaileyales [Independent clade] | Austobaileyaceae [5]<br>Schisandraceae [7]<br>Trimeniaceae [6] | 1<br>3<br>1 | 1<br>85<br>8 | <br>2<br> | | <br>2<br> | | | |
| | **All Austrobaileyales** | **3** | **5** | **94** | **2 (2.13%)** | | **2** | | **0%** | **0%** |
| [D] | Canellales [Magnolids] | Canellaceae [8]<br>Winteraceae [9] | 5<br>5 | 23<br>65 | <br>1 | | <br>1 | | | |
| | **All Canellales** | **2** | **10** | **88** | **1 (1.14%)** | | **1** | | **0%** | **0%** |
| [E] | Piperales [Mangolids] | Aristolochiaceae [12]<br>Piperaceae [11]<br>Saururaceae [10] | 7<br>5<br>4 | 500<br>3700<br>6 | 3<br>3<br> | 1<br>1<br> | 2<br>2<br> | | | |
| | **All Piperales** | **3** | **16** | **4206** | **6 (0.14%)** | **2** | **4** | | **0.048%** | **33.3%** |
| [F] | Magnoliales [Magnolids] | Annonaceae [18]<br>Degeneriaceae [15]<br>Eupomatiaceae [17]<br>Himantandraceae [16]<br>Magnoliaceae [14]<br>Myristicaceae [13] | 105<br>1<br>1<br>1<br>2<br>21 | 2500<br>2<br>3<br>2<br>294<br>520 | 6<br><br><br><br>8<br> | <br><br><br><br>5<br> | 6<br><br><br><br>3<br> | | | |

| | | | | | | | | | | |
|---|---|---|---|---|---|---|---|---|---|---|
| | **All Magnoliales** | **6** | **131** | **3321** | **14 (0.42%)** | **5** | **9** | | **0.151%** | **35.7%** |
| [G] | Laurales [Magnolids] | Anthereospermataceae [22] | 6 | 16 | | | | | | |
| | | Calycathaceae [19] | 3 | 10 | 2 | | 2 | | | |
| | | Gomortegaceae [21] | 1 | 1 | | | | | | |
| | | Hernandiaceae [23] | 5 | 58 | | | | | | |
| | | Lauraceae [25] | 45 | 2850 | 3 | 1 | 2 | | | |
| | | Monimiaceae [24] | 24 | 217 | | | | | | |
| | | Siparunaceae [20] | 2 | 75 | | | | | | |
| | **All Laurales** | **7** | **86** | **3227** | **5 (0.16%)** | **1** | **4** | | **0.031%** | **20.0%** |
| [H] | Chloranthales [Independent clade] | Chloranthaceae [26] | 4 | 77 | | | | | | **no data** |
| [I] | Acorales [Monocots] | Acoraceae [27] | **1** | **2** | **1 (50.0%)** | | **1** | | **0%** | **0%** |
| [J] | Alismatales [Monocots] | Alismataceae [30] | 17 | 115 | 5 | | 5 | | | |
| | | Aponogetonaceae [34] | 1 | 56 | | | | | | |
| | | Araceae [28] | 114 | 3750 | 20 | | 20 | | | |
| | | Butomaceae [31] | 1 | 1 | 1 | 1 | | | | |
| | | Cymodoceaceae [41] | 5 | 17 | | | | | | |
| | | Hydrocharitaceae [32] | 16 | 135 | | | | | | |
| | | Juncaginaceae [35] | 3 | 34 | | | | | | |
| | | Maundiaceae [36] | 1 | 1 | | | | | | |
| | | Posidoniaceae [39] | 1 | 9 | | | | | | |
| | | Potamogetonaceae [38] | 6 | 110 | | | | | | |
| | | Ruppiaceae [40] | 1 | 8 | | | | | | |
| | | Scheuchzeriaceae [33] | 1 | 1 | | | | | | |
| | | Tofieldiaceae [29] | 4 | 28 | 2 | 1 | 1 | | | |
| | | Zosteraceae [37] | 2 | 22 | | | | | | |
| | **All Alismatales** | **14** | **173** | **4287** | **28 (0.65%)** | **2** | **26** | | **0.047%** | **7.1%** |
| [K] | Petrosaviales [Monocots] | Petrosaviaceae [42] | 2 | 4 | | | | | | **no data** |
| [L] | Dioscoreales [Monocots] | Burmanniaceae [44] | 8 | 99 | | | | | | |
| | | Dioscoreaceae [45] | 9 | 715 | 2 | | 2 | | | |
| | | Nartheciaceae [43] | 5 | 38 | 1 | | 1 | | | |
| | **All Dioscoreales** | **3** | **22** | **852** | **3 (0.35%)** | | **3** | | **0%** | **0%** |
| [M] | Pandanales [Monocots] | Cyclanthaceae [49] | 12 | 230 | | | | | | |
| | | Pandanaceae [50] | 5 | 982 | | | | | | |
| | | Stemonaceae [48] | 4 | 37 | | | | | | |
| | | Triuridaceae [46] | 9 | 55 | | | | | | |
| | | Velloziaceae [47] | 5 | 306 | | | | | | |
| | **All Pandanales** | **5** | **35** | **1610** | | | | | | **no data** |
| [N] | Liliales [Monocots] | Alstroemeriaceae [55] | 4 | 254 | 4 | | 4 | | | |
| | | Campynemataceae [51] | 2 | 4 | | | | | | |

| | | | | | | | | | | |
|---|---|---|---|---|---|---|---|---|---|---|
| | | Colchicaceae [56] | 15 | 285 | 7 | 1 | 6 | | | |
| | | Corsiaceae [52] | 3 | 27 | | | | | | |
| | | Liliaceae [60] | 15 | 705 | 38 | 6 | 32 | | | |
| | | Melanthiaceae [53] | 17 | 173 | 12 | 11 | 1 | | | |
| | | Petermanniaceae [54] | 1 | 1 | | | | | | |
| | | Philesiaceae [57] | 2 | 2 | 1 | | 1 | | | |
| | | Ripogonaceae [58] | 1 | 6 | | | | | | |
| | | Smilacaceae [59] | 1 | 255 | 1 | | 1 | | | |
| | **All Liliales** | **10** | **61** | **1712** | **63 (3.68%)** | **18** | **45** | | **1.051%** | **28.6%** |
| [O] | Asparagales | Amaryllidaceae [73] | 75 | 1600 | 41 | 8 | 31 | 2 | | |
| | [Monocots] | Asparagaceae [74] | 114 | 2900 | 86 | 24 | 62 | | | |
| | | Asphodelaceae [72] | 39 | 900 | 15 | | 15 | | | |
| | | Asteliaceae [64] | 3 | 37 | 1 | | 1 | | | |
| | | Blandfordiaceae [63] | 1 | 4 | 2 | 2 | | | | |
| | | Boryaceae [62] | 2 | 12 | | | | | | |
| | | Doryanthaceae [67] | 1 | 2 | 1 | 1 | | | | |
| | | Hypoxidaceae [66] | 4 | 159 | 1 | 1 | | | | |
| | | Iridaceae [70] | 66 | 2244 | 64 | 4 | 60 | | | |
| | | Ixioliriaceae [68] | 1 | 4 | | | | | | |
| | | Lanariaceae [65] | 1 | 1 | | | | | | |
| | | Orchidaceae [61] | 736 | 28000 | 1 | | 1 | | | |
| | | Tecophilaeaceae [69] | 9 | 27 | | | | | | |
| | | Xeronemataceae [71] | 1 | 2 | 1 | | 1 | | | |
| | **All Asparagales** | **14** | **1053** | **35892** | **213 (0.59%)** | **40** | **171** | **2** | **0.111%** | **18.8%** |
| [P] | Arecales | Arecaceae [76] | 181 | 2600 | 22 | 2 | 20 | | | |
| | [Monocots] | Dasypogonaceae [75] | 4 | 16 | | | | | | |
| | **All Arecales** | **2** | **185** | **2616** | **22 (0.84%)** | **2** | **20** | | **0.076%** | **9.1%** |
| [Q] | Commelinales | Commelinaceae [78] | 41 | 731 | 11 | 4 | 6 | 1 | | |
| | [Monocots] | Haemodoraceae [81] | 14 | 102 | 5 | | 5 | | | |
| | | Hanguanaceae [77] | 1 | 12 | | | | | | |
| | | Philydraceae [79] | 3 | 6 | | | | | | |
| | | Pontederiaceae [80] | 6 | 34 | | | | | | |
| | **All Commelinales** | **5** | **65** | **885** | **16 (1.81%)** | **4** | **11** | **1** | **0.452%** | **25.0%** |
| [R] | Zingiberales | Cannaceae [86] | 1 | 10 | 1 | 1 | | | | |
| | [Monocots] | Costaceae [88] | 7 | 143 | 1 | | 1 | | | |
| | | Heliconiaceae [84] | 1 | 194 | | | | | | |
| | | Lowiaceae [83] | 1 | 18 | | | | | | |
| | | Marantaceae [87] | 29 | 570 | 1 | | 1 | | | |
| | | Musaceae [85] | 3 | 91 | 3 | | 3 | | | |
| | | Strelitziaceae [82] | 3 | 7 | 3 | | 3 | | | |
| | | Zingiberaceae [89] | 50 | 1600 | 7 | | 7 | | | |
| | **All Zingiberales** | **8** | **95** | **2633** | **16 (0.61%)** | **1** | **15** | | **0.038%** | **6.3%** |
| [S] | Poales | Bromeliaceae [91] | 51 | 3475 | 8 | 1 | 7 | | | |

| | | | | | | | | | | |
|---|---|---|---|---|---|---|---|---|---|---|
| | [Monocots] | Cyperaceae [98] | 9 | 5500 | 34 | 11 | 23 | | | |
| | | Ecdeiocoleaceae [102] | 2 | 3 | | | | | | |
| | | Eriocaulaceae [94] | 7 | 1207 | | | | | | |
| | | Flagellariaceae [100] | 1 | 4 | | | | | | |
| | | Joinvilleaceae [101] | 1 | 4 | | | | | | |
| | | Juncaceae [97] | 8 | 464 | 2 | | 2 | | | |
| | | Mayacaceae [95] | 1 | 6 | | | | | | |
| | | Poaceae [103] | 780 | 12000 | 146 | 18 | 126 | 2 | | |
| | | Rapateaceae [92] | 16 | 94 | | | | | | |
| | | Restionaceae [99] | 51 | 572 | 2 | | 2 | | | |
| | | Thurniaceae [96] | 2 | 4 | | | | | | |
| | | Typhaceae [90] | 2 | 51 | 7 | 1 | 6 | | | |
| | | Xyridaceae [93] | 5 | 399 | 1 | | 1 | | | |
| | **All Poales** | **15** | **936** | **23783** | **200 (0.84%)** | **31** | **167** | **2** | **0.130%** | **15.5%** |
| [T] | Ceratophyllales [Sister of Eudicots] | Ceratophyllaceae [104] | 1 | 4 | | | | | | **no data** |
| [U] | Ranunculales [Eudicots] | Berberidaceae [110] | 14 | 700 | 17 | 2 | 15 | | | |
| | | Circaeasteraceae [107] | 2 | 2 | | | | | | |
| | | Eupteleaceae [105] | 1 | 2 | | | | | | |
| | | Lardizabalaceae [108] | 7 | 40 | 3 | | 3 | | | |
| | | Menispermaceae [109] | 68 | 440 | 1 | | 1 | | | |
| | | Papaveraceae [106] | 42 | 775 | 54 | 4 | 50 | | | |
| | | Ranunculaceae [111] | 43 | 2346 | 169 | 57 | 112 | | | |
| | **All Ranunculales** | **7** | **177** | **4305** | **244 (5.67%)** | **63** | **181** | | **1.463%** | **25.8%** |
| [V] | Proteales [Eudicots] | Nelumbonaceae [113] | 1 | 3 | 1 | | 1 | | | |
| | | Platanaceae [114] | 1 | 8 | 1 | | 1 | | | |
| | | Proteaceae [115] | 83 | 1660 | 26 | | 26 | | | |
| | | Sabiaceae [112] | 3 | 66 | | | | | | |
| | **All Proteales** | **4** | **88** | **1737** | **28 (1.61%)** | | **28** | | **0%** | **0%** |
| [W] | Trochodendrales [Eudicots] | Trochodendraceae [116] | 2 | 2 | | | | | | **no data** |
| [X] | Buxales [Eudicots] | Buxaceae [117] | 6 | 123 | | | | | | **no data** |
| [Y] | Gunnerales [Core Eudicots] | Gunneraceae [119] | 1 | 63 | 4 | | 4 | | | |
| | | Myrothamnaceae [118] | 1 | 2 | | | | | | |
| | **All Gunnerales** | **2** | **2** | **65** | **4 (6.15%)** | | **4** | | **0%** | **0%** |
| [Z] | Dilleniales [Core Eudicots] | Dilleniaceae [120] | **11** | **430** | **2 (0.47%)** | | **2** | | **0%** | **0%** |
| [AA] | Saxifragales | Altingiaceae [123] | 1 | 15 | 1 | | 1 | | | |

| | | | | | | | | | | |
|---|---|---|---|---|---|---|---|---|---|---|
| | [Eudicots – Superrosids] | Aphanopetalaceae [131] | 1 | 2 | | | | | | |
| | | Cercidiphyllaceae [125] | 1 | 2 | 1 | | 1 | | | |
| | | Crassulaceae [130] | 35 | 1400 | 20 | | 20 | | | |
| | | Cynomoriaceae [135] | 1 | 2 | | | | | | |
| | | Daphniphyllaceae [126] | 1 | 30 | | | | | | |
| | | Grossulariaceae [128] | 1 | 150 | 2 | | 2 | | | |
| | | Haloragaceae [134] | 19 | 145 | | | | | | |
| | | Hamamelidaceae [124] | 26 | 86 | 4 | | 4 | | | |
| | | Iteaceae [127] | 2 | 18 | | | | | | |
| | | Paeoniaceae [122] | 1 | 33 | 7 | 1 | 6 | | | |
| | | Penthoraceae [133] | 1 | 2 | | | | | | |
| | | Peridiscaceae [121] | 4 | 12 | | | | | | |
| | | Saxifragaceae [129] | 33 | 640 | 39 | | 39 | | | |
| | | Tetracarpaeaceae [132] | 1 | 1 | | | | | | |
| | **All Saxifragales** | **15** | **128** | **2538** | **74 (2.92%)** | **1** | **73** | | **0.039%** | **1.4%** |
| [BB] | Vitales [Eudicots – Rosids] | Vitaceae [136] | **14** | **910** | **13 (1.43%)** | **1** | **12** | | **0.110%** | **7.7%** |
| [CC] | Zygophyllales [Eudicots – Rosids] | Krameriaceae [137] | 1 | 18 | | | | | | |
| | | Zygophyllaceae [138] | 22 | 285 | 2 | 1 | 1 | | | |
| | **All Zygophyllales** | **2** | **23** | **303** | **2 (0.66%)** | **1** | **1** | | **0.330%** | **50.0%** |
| [DD] | Fabales [Eudicots – Rosids] | Fabaceae [140] | 751 | 19500 | 200 | 6 | 194 | | | |
| | | Polygalaceae [142] | 21 | 900 | 2 | | 2 | | | |
| | | Quillajaceae [139] | 1 | 3 | | | | | | |
| | | Surianaceae [141] | 5 | 8 | | | | | | |
| | **All Fabales** | **4** | **778** | **20411** | **202 (0.99%)** | **6** | **196** | | **0.029%** | **3.0%** |
| [EE] | Rosales [Eudicots – Rosids] | Barbeyaceae [144] | 1 | 1 | | | | | | |
| | | Cannabaceae [149] | 8 | 100 | 5 | | 5 | | | |
| | | Dirachmaceae [145] | 1 | 2 | | | | | | |
| | | Elaeagnaceae [146] | 3 | 60 | 4 | | 4 | | | |
| | | Moraceae [150] | 38 | 1180 | 12 | 1 | 11 | | | |
| | | Rhamnaceae [147] | 55 | 950 | 16 | | 16 | | | |
| | | Rosaceae [143] | 91 | 2950 | 183 | 8 | 175 | | | |
| | | Ulmaceae [148] | 7 | 45 | 3 | | 3 | | | |
| | | Urticaceae [151] | 53 | 2625 | 8 | | 8 | | | |
| | **All Rosales** | **9** | **257** | **7913** | **231 (2.92%)** | **9** | **222** | | **0.114%** | **3.9%** |
| [FF] | Fagales [Eudicots – Rosids] | Betulaceae [158] | 6 | 167 | 39 | 2 | 35 | 2 | | |
| | | Casuarinaceae [156] | 4 | 91 | 2 | | 2 | | | |
| | | Fagaceae [153] | 8 | 927 | 3 | | 3 | | | |
| | | Juglandaceae [155] | 9 | 50 | | | | | | |
| | | Myricaceae [154] | 3 | 57 | 2 | | 2 | | | |
| | | Nothofagaceae [152] | 1 | 48 | 3 | | 3 | | | |
| | | Ticodendraceae [157] | 1 | 1 | | | | | | |
| | **All Fagales** | **7** | **32** | **1341** | **49 (3.65%)** | **2** | **45** | **2** | **0.149%** | **4.1%** |

| | | | | | | | | | | |
|---|---|---|---|---|---|---|---|---|---|---|
| [GG] | Cucurbitales | Anisophyllaceae [160] | 4 | 71 | | | | | | |
| | [Eudicots – Rosids] | Apodanthaceae [159] | 2 | 10 | | | | | | |
| | | Begoniaceae [166] | 2 | 1825 | 5 | | 5 | | | |
| | | Coriariaceae [162] | 1 | 14 | 1 | | 1 | | | |
| | | Corynocarpaceae [161] | 1 | 5 | | | | | | |
| | | Cucurbitaceae [163] | 95 | 965 | 14 | 5 | 9 | | | |
| | | Datiscaceae [165] | 1 | 3 | 1 | 1 | | | | |
| | | Tetramelaceae [164] | 2 | 2 | | | | | | |
| | **All Cucurbitales** | **8** | **110** | **2895** | **21 (0.73%)** | **6** | **15** | | **0.207%** | **28.6%** |
| [HH] | Celastrales | Celastraceae [168] | 96 | 1350 | 9 | | 9 | | | |
| | [Eudicots – Rosids] | Lepidobotryaceae [167] | 2 | 2 | | | | | | |
| | **All Celastrales** | **2** | **98** | **1352** | **9 (0.67%)** | | **9** | | **0%** | **0%** |
| [II] | Oxalidales | Brunelliaceae [175] | 1 | 60 | | | | | | |
| | [Eudicots – Rosids] | Cephalotaceae [174] | 1 | 1 | | | | | | |
| | | Connaraceae [170] | 12 | 180 | | | | | | |
| | | Cunoniaceae [172] | 27 | 330 | 6 | | 6 | | | |
| | | Elaeocarpaceae [173] | 12 | 615 | 6 | 1 | 5 | | | |
| | | Huaceae [169] | 2 | 4 | | | | | | |
| | | Oxalidaceae [171] | 5 | 570 | | | | | | |
| | **All Oxalidales** | **7** | **60** | **1760** | **12 (0.68%)** | **1** | **11** | | **0.057%** | **8.3%** |
| [JJ] | Malpighiales | Achariaceae [199] | 32 | 155 | | | | | | |
| | [Eudicots – Rosids] | Balanopacaceae [193] | 1 | 9 | | | | | | |
| | | Bonnetiaceae [182] | 3 | 35 | | | | | | |
| | | Calophyllaceae [184] | 14 | 475 | 2 | | 2 | | | |
| | | Caryocaraceae [187] | 2 | 26 | | | | | | |
| | | Centroplacaceae [190] | 2 | 6 | | | | | | |
| | | Chrysobalanaceae [197] | 18 | 533 | | | | | | |
| | | Clusiaceae [183] | 13 | 750 | | | | | | |
| | | Ctenolophonaceae [178] | 1 | 2 | | | | | | |
| | | Dichapetalaceae [195] | 3 | 170 | | | | | | |
| | | Elatinaceae [191] | 2 | 35 | | | | | | |
| | | Erythroxylaceae [180] | 4 | 242 | | | | | | |
| | | Euphorbiaceae [207] | 209 | 6252 | 23 | 1 | 22 | | | |
| | | Euphroniaceae [196] | 1 | 3 | | | | | | |
| | | Goupiaceae [201] | 1 | 2 | | | | | | |
| | | Humiriaceae [198] | 5 | 56 | | | | | | |
| | | Hypericaceae [186] | 6 | 590 | 17 | 2 | 15 | | | |
| | | Irvingiaceae [177] | 3 | 13 | | | | | | |
| | | Ixonanthaceae [209] | 3 | 17 | | | | | | |
| | | Lacistemataceae [203] | 2 | 14 | | | | | | |
| | | Linaceae [208] | 10 | 255 | 5 | | 5 | | | |
| | | Lophopyxidaceae [188] | 1 | 1 | | | | | | |
| | | Malphigiaceae [192] | 73 | 1315 | 2 | | 2 | | | |

| | | | | | | | | | | |
|---|---|---|---|---|---|---|---|---|---|---|
| | | Ochnaceae [181] | 32 | 550 | | | | | | |
| | | Pandaceae [176] | 3 | 17 | | | | | | |
| | | Passifloraceae [202] | 29 | 980 | 14 | | 14 | | | |
| | | Peraceae [205] | 5 | 127 | | | | | | |
| | | Phyllanthaceae [211] | 57 | 2050 | 1 | | 1 | | | |
| | | Picrodendraceae [210] | 25 | 96 | | | | | | |
| | | Podostemaceae [185] | 46 | 300 | | | | | | |
| | | Putranjivaceae [189] | 2 | 216 | | | | | | |
| | | Rafflesiaceae [206] | 3 | 25 | | | | | | |
| | | Rhizophoraceae [179] | 15 | 147 | | | | | | |
| | | Salicaceae [204] | 56 | 1220 | 3 | | 3 | | | |
| | | Trigoniaceae [194] | 5 | 28 | | | | | | |
| | | Violaceae [200] | 31 | 980 | 13 | | 13 | | | |
| | **All Malpigiales** | **36** | **717** | **17692** | **80 (0.45%)** | **3** | **77** | | **0.017%** | **3.8%** |
| [KK] | Geraniales | Francoaceae [213] | 8 | 37 | 1 | | 1 | | | |
| | [Eudicots – Rosids] | Geraniaceae [212] | 5 | 830 | 31 | | 31 | | | |
| | **All Gerianales** | **2** | **13** | **867** | **32 (3.69%)** | | **32** | | **0%** | **0%** |
| [LL] | Myrtales | Alzateaceae [221] | 1 | 1 | | | | | | |
| | [Eudicots – Rosids] | Combretaceae [214] | 10 | 530 | | | | | | |
| | | Crypteroniaceae [220] | 3 | 13 | | | | | | |
| | | Lythraceae [215] | 27 | 620 | 6 | | 6 | | | |
| | | Melastomataceae [219] | 165 | 5115 | 5 | | 5 | | | |
| | | Myrtaceae [218] | 132 | 5950 | 98 | 2 | 96 | | | |
| | | Onagraceae [216] | 22 | 656 | 25 | | 25 | | | |
| | | Penaeaceae [222] | 9 | 32 | 1 | | 1 | | | |
| | | Vochysiaceae [217] | 7 | 217 | | | | | | |
| | **All Myrtales** | **9** | **376** | **13134** | **135 (1.03%)** | **2** | **133** | | **0.015%** | **1.5%** |
| [MM] | Crossosomatales | Asphloaceae [223] | 1 | 1 | | | | | | |
| | [Eudicots – Rosids] | Crossosomataceae [229] | 4 | 10 | | | | | | |
| | | Geissolomataceae [224] | 1 | 1 | | | | | | |
| | | Guamatelaceae [227] | 1 | 1 | | | | | | |
| | | Stachyuraceae [228] | 1 | 8 | | | | | | |
| | | Staphyleaceae [226] | 2 | 45 | 1 | | 1 | | | |
| | | Strasburgeriaceae [225] | 2 | 2 | | | | | | |
| | **All Crossomatales** | **7** | **12** | **68** | **1 (1.47%)** | | **1** | | **0%** | **0%** |
| [NN] | Picramniales [Eudicots –Rosids] | Picramniaceae [230] | 3 | 49 | | | | | | **no data** |
| [OO] | Huerteales | Dipentodontaceae [234] | 2 | 20 | | | | | | |
| | [Eudicots – Rosids] | Gerrardinaceae [231] | 1 | 2 | | | | | | |
| | | Petenaeceae [232] | 1 | 1 | | | | | | |
| | | Tapisciaceae [233] | 2 | 6 | | | | | | |
| | **All Huerteales** | **4** | **6** | **29** | | | | | | **no data** |

| | | | | | | | | | | |
|---|---|---|---|---|---|---|---|---|---|---|
| [PP] | Sapindales | Anacardiaceae [239] | 83 | 860 | 13 | 2 | 11 | | | |
| | [Eudicots – Rosids] | Burseraceae [238] | 19 | 615 | | | | | | |
| | | Biebersteiniaceae [235] | 1 | 4 | | | | | | |
| | | Kirkiaceae [237] | 1 | 6 | | | | | | |
| | | Meliaceae [243] | 53 | 600 | 18 | 3 | 12 | 3 | | |
| | | Nitrariaceae [236] | 3 | 19 | 1 | | 1 | | | |
| | | Rutaceae [241] | 148 | 2070 | 23 | 3 | 20 | | | |
| | | Sapindaceae [240] | 142 | 1860 | 26 | | 26 | | | |
| | | Simaroubaceae [242] | 22 | 108 | 1 | | 1 | | | |
| | **All Sapindales** | **9** | **472** | **6142** | **82 (1.34%)** | **8** | **71** | **3** | **0.130%** | **9.8%** |
| [QQ] | Malvales | Bixaceae [250] | 4 | 23 | 2 | | 2 | | | |
| | [Eudicots –Rosids] | Cistaceae [251] | 9 | 170 | 16 | 1 | 15 | | | |
| | | Cytinaceae [244] | 2 | 10 | | | | | | |
| | | Dipterocarpaceae [253] | 16 | 695 | | | | | | |
| | | Malvaceae [247] | 244 | 4225 | 72 | 6 | 66 | | | |
| | | Muntingiaceae [245] | 3 | 3 | | | | | | |
| | | Neuradaceae [246] | 3 | 10 | | | | | | |
| | | Sarcolaenaceae [252] | 10 | 71 | | | | | | |
| | | Sphaerosepalaceae [248] | 2 | 18 | | | | | | |
| | | Thymelaeaceae [249] | 46 | 913 | 5 | 1 | 4 | | | |
| | **All Malvales** | **10** | **339** | **6138** | **95 (1.55%)** | **8** | **87** | | **0.130%** | **8.4%** |
| [RR] | Brassicales | Akaniaceae [254] | 2 | 2 | | | | | | |
| | [Eudicots – Rosids] | Bataceae [261] | 1 | 2 | | | | | | |
| | | Brassicaceae [270] | 328 | 3628 | 88 | 9 | 79 | | | |
| | | Capparaceae [268] | 30 | 324 | 6 | | 6 | | | |
| | | Caricaceae [257] | 6 | 35 | 3 | | 3 | | | |
| | | Cleomaceae [269] | 1 | 346 | | | | | | |
| | | Emblingiaceae [263] | 1 | 1 | | | | | | |
| | | Gyrostemonaceae [266] | 4 | 20 | | | | | | |
| | | Koeberliniaceae [260] | 1 | 2 | | | | | | |
| | | Limnanthaceae [258] | 2 | 8 | 6 | 6 | | | | |
| | | Moringaceae [256] | 1 | 13 | | | | | | |
| | | Pentadiplandraceae [265] | 1 | 1 | | | | | | |
| | | Resedaceae [267] | 12 | 107 | 3 | | 2 | 1 | | |
| | | Salvadoraceae [262] | 3 | 11 | | | | | | |
| | | Setchellanthaceae [259] | 1 | 1 | | | | | | |
| | | Tovariaceae [264] | 1 | 2 | | | | | | |
| | | Tropaeolaceae [255] | 1 | 94 | 3 | | 3 | | | |
| | **All Brassicales** | **17** | **396** | **4596** | **109 (2.37%)** | **15** | **93** | **1** | **0.326%** | **13.8%** |
| [SS] | Berberidopsidales | Aextoxicaceae [271] | 1 | 1 | | | | | | |
| | [Eudicots – Superasterids] | Berberidopsidaceae [272] | 2 | 3 | | | | | | |
| | **All** | **2** | **3** | **4** | | | | | | **no data** |

| | | | | | | | | | | |
|---|---|---|---|---|---|---|---|---|---|---|
| | **Berberidopsidales** | | | | | | | | | |
| [TT] | Santalales | Balanophoraceae [275] | 17 | 44 | | | | | | |
| | [Eudicots – | Loranthaceae [279] | 76 | 1050 | 1 | | 1 | | | |
| | Superasterids] | Misodendraceae [277] | 1 | 8 | | | | | | |
| | | Olacaceae [273] | 29 | 180 | | | | | | |
| | | Opiliaceae [274] | 11 | 33 | | | | | | |
| | | Santalaceae [276] | 43 | 1000 | 1 | | 1 | | | |
| | | Schoepfiaceae [278] | 3 | 58 | | | | | | |
| | **All Santalales** | **7** | **180** | **2373** | **2 (0.08%)** | | **2** | | **0%** | **0%** |
| [UU] | Caryophyllales | Achatocarpaceae [296] | 2 | 11 | | | | | | |
| | [Eudicots – | Aizoaceae [304] | 121 | 1900 | 27 | 2 | 25 | | | |
| | Superasterids] | Amaranthaceae [297] | 165 | 2040 | 66 | 27 | 38 | 1 | | |
| | | Anacampserotaceae [316] | 3 | 36 | 1 | | 1 | | | |
| | | Ancistrocladaceae [288] | 1 | 21 | | | | | | |
| | | Asteropeiaceae [292] | 1 | 8 | | | | | | |
| | | Barbeuiaceae [302] | 1 | 1 | | | | | | |
| | | Basellaceae [312] | 4 | 19 | 1 | | 1 | | | |
| | | Cactaceae [317] | 127 | 1750 | 11 | | 11 | | | |
| | | Caryophyllaceae [295] | 81 | 2625 | 130 | 42 | 84 | 4 | | |
| | | Didiereaceae [311] | 7 | 22 | | | | | | |
| | | Dioncophyllaceae [287] | 3 | 3 | | | | | | |
| | | Droseraceae [284] | 3 | 180 | 9 | | 9 | | | |
| | | Drosphyllaceae [286] | 1 | 1 | | | | | | |
| | | Frankeniaceae [280] | 1 | 90 | | | | | | |
| | | Gisekiaceae [303] | 1 | 8 | 1 | | 1 | | | |
| | | Halophytaceae [313] | 1 | 1 | | | | | | |
| | | Kewaceae [301] | 1 | 8 | | | | | | |
| | | Limeaceae [299] | 1 | 21 | | | | | | |
| | | Lophiocarpaceae [300] | 2 | 6 | 1 | | 1 | | | |
| | | Macarthuriaceae [293] | 1 | 10 | | | | | | |
| | | Microteaceae [294] | 1 | 9 | | | | | | |
| | | Molluginaceae [309] | 9 | 80 | 2 | | 2 | | | |
| | | Montiaceae [310] | 14 | 230 | 4 | | 4 | | | |
| | | Nepenthaceae [285] | 1 | 150 | 2 | | 2 | | | |
| | | Nyctaginaceae [308] | 31 | 400 | 1 | | 1 | | | |
| | | Petiveriaceae [306] | 9 | 20 | 4 | | 4 | | | |
| | | Physenaceae [291] | 1 | 2 | | | | | | |
| | | Phytolaccaceae [305] | 5 | 33 | 6 | 1 | 5 | | | |
| | | Plumbaginaceae [282] | 30 | 725 | 23 | 8 | 15 | | | |
| | | Polygonaceae [283] | 48 | 1200 | 37 | 1 | 36 | | | |
| | | Portulacaceae [315] | 1 | 115 | 1 | | 1 | | | |
| | | Rhabdodendraceae [289] | 1 | 3 | | | | | | |

| | | | | | | | | | | |
|---|---|---|---|---|---|---|---|---|---|---|
| | | Sarcobataceae [307] | 1 | 2 | | | | | | |
| | | Simmondsiaceae [290] | 1 | 1 | 1 | | 1 | | | |
| | | Stegnospermataceae [298] | 1 | 4 | | | | | | |
| | | Talinaceae [314] | 2 | 28 | 2 | | 2 | | | |
| | | Tamaricaceae [281] | 4 | 78 | | | | | | |
| | **All Caryophyllales** | **38** | **688** | **11841** | **330 (2.79%)** | **81** | **244** | **5** | **0.684%** | **24.6%** |
| [VV] | Cornales [Eudicots – Asterids] | Cornaceae [324] | 2 | 85 | 10 | | 10 | | | |
| | | Curtisiaceae [322] | 1 | 1 | | | | | | |
| | | Grubbiaceae [323] | 1 | 3 | | | | | | |
| | | Hydrangeaceae [320] | 9 | 223 | 4 | | 4 | | | |
| | | Hydrostachyaceae [319] | 1 | 22 | | | | | | |
| | | Loasaceae [321] | 20 | 308 | 7 | 2 | 5 | | | |
| | | Nyssaceae [318] | 5 | 37 | 2 | 1 | 1 | | | |
| | **All Cornales** | **7** | **39** | **679** | **23 (3.39%)** | **3** | **20** | | **0.442%** | **13.0%** |
| [WW] | Ericales [Eudicots – Asterids] | Actinidiaceae [342] | 3 | 360 | 3 | | 3 | | | |
| | | Balsaminaceae [325] | 2 | 1000 | 3 | 2 | 1 | | | |
| | | Clethraceae [343] | 2 | 75 | 1 | 1 | | | | |
| | | Cyrillaceae [344] | 2 | 2 | | | | | | |
| | | Diapensiaceae [338] | 5 | 12 | | | | | | |
| | | Ebenaceae [334] | 4 | 800 | 6 | 1 | 5 | | | |
| | | Ericaceae [345] | 124 | 4250 | 55 | 1 | 54 | | | |
| | | Fouquieriaceae [328] | 1 | 11 | 1 | | 1 | | | |
| | | Lecythidaceae [330] | 25 | 355 | 2 | 1 | 1 | | | |
| | | Marcgraviaceae [326] | 7 | 120 | | | | | | |
| | | Mitrastemonaceae [346] | 1 | 2 | | | | | | |
| | | Pentaphylaceae [332] | 12 | 330 | 1 | | 1 | | | |
| | | Polemoniaceae [329] | 26 | 350 | 17 | 1 | 16 | | | |
| | | Primulaceae [335] | 53 | 2790 | 72 | 2 | 69 | 1 | | |
| | | Roridulaceae [341] | 1 | 2 | | | | | | |
| | | Sapotaceae [333] | 54 | 1273 | | | | | | |
| | | Sarraceniaceae [340] | 3 | 34 | 4 | | 4 | | | |
| | | Sladeniaceae [331] | 2 | 3 | | | | | | |
| | | Styracaceae [339] | 11 | 160 | 2 | | 2 | | | |
| | | Symplocaceae [337] | 2 | 260 | | | | | | |
| | | Tetrameristaceae [327] | 3 | 5 | | | | | | |
| | | Theaceae [336] | 9 | 240 | 2 | | 2 | | | |
| | **All Ericales** | **22** | **352** | **12435** | **169 (1.36%)** | **9** | **159** | **1** | **0.072%** | **5.3%** |
| [XX] | Icacinales [Eudicots – Asterids] | Icacinaceae [348] | 25 | 165 | | | | | | |
| | | Oncothecaceae [347] | 1 | 2 | | | | | | |
| | **All Icacinalaes** | **2** | **26** | **167** | | | | | | **no data** |
| [YY] | Metteniusales [Eudicots – Asterids] | Metteniusaceae [349] | **11** | **50** | **1 (2.00%)** | | **1** | | **0%** | **0%** |

| | | | | | | | | | | |
|---|---|---|---|---|---|---|---|---|---|---|
| [ZZ] | Garryales<br>[Eudicots – Asterids] | Eucommiaceae [350]<br>Garryaceae [351] | 1<br>2 | 1<br>25 | 1<br>2 | | 1<br>2 | | | |
| | **All Garryales** | **2** | **3** | **26** | **3 (11.5%)** | | **3** | | **0%** | **0%** |
| [AAA] | Gentianales<br>[Eudicots – Asterids] | Apocynaceae [356]<br>Gelsemiaceae [355]<br>Gentianaceae [353]<br>Loganiaceae [354]<br>Rubiaceae [352] | 366<br>3<br>102<br>15<br>590 | 5100<br>11<br>1735<br>390<br>13620 | 32<br><br>29<br><br>22 | 3<br><br><br><br>1 | 29<br><br>29<br><br>21 | | | |
| | **All Gentianales** | **5** | **1076** | **20856** | **83 (0.40%)** | **4** | **79** | | **0.019%** | **4.8%** |
| [BBB] | Boraginales<br>[Eudicots – Asterids] | Boraginaceae [357] | **135** | **2535** | **61 (2.41%)** | **6** | **54** | **1** | **0.237%** | **9.8%** |
| [CCC] | Vahliales<br>[Eudicots – Asterids] | Vahliaceae [358] | 1 | 8 | | | | | | **no data** |
| [DDD] | Solanales<br>[Eudicots – Asterids] | Convolvulaceae [359]<br>Hydroleaceae [363]<br>Montiniaceae [361]<br>Solanaceae [360]<br>Sphenocleaceae [362] | 53<br>1<br>3<br>100<br>1 | 1660<br>12<br>5<br>2600<br>2 | 11<br><br>2<br>134<br> | <br><br><br>27<br> | 11<br><br>2<br>105<br> | <br><br><br>2<br> | | |
| | **All Solanales** | **5** | **158** | **4279** | **147 (3.44%)** | **27** | **118** | **2** | **0.631%** | **18.4%** |
| [EEE] | Lamiales<br>[Eudicots – Asterids] | Acanthaceae [377]<br>Bignoniaceae [378]<br>Byblidaceae [374]<br>Calceolariaceae [368]<br>Carlemanniaceae [365]<br>Gesneriaceae [369]<br>Lamiaceae [383]<br>Lentibulariaceae [379]<br>Linderniaceae [373]<br>Martyniaceae [375]<br>Mazaceae [384]<br>Oleaceae [366]<br>Orabanchaceae [387]<br>Paulowniaceae [386]<br>Pedaliaceae [376]<br>Phyrmaceae [385]<br>Plantaginaceae [370]<br>Plocospermataceae [364]<br>Schlegeliaceae [380]<br>Scrophulariaceae [371]<br>Stilbaceae [372]<br>Tetrachondraceae [367]<br>Thomandersiaceae [381]<br>Verbenaceae [382] | 210<br>82<br>1<br>2<br>2<br>152<br>241<br>3<br>23<br>5<br>3<br>26<br>98<br>3<br>13<br>13<br>94<br>1<br>4<br>62<br>8<br>2<br>1<br>32 | 4000<br>870<br>7<br>271<br>5<br>3540<br>7530<br>316<br>220<br>16<br>33<br>790<br>1960<br>8<br>75<br>136<br>1900<br>1<br>37<br>1830<br>40<br>3<br>6<br>1000 | 6<br>17<br><br>3<br><br>21<br>141<br>2<br>1<br>1<br><br>11<br>10<br>2<br>2<br>5<br>101<br><br><br>26<br><br><br><br>17 | <br>2<br><br><br><br><br>13<br><br><br><br><br><br>1<br><br><br><br>15<br><br><br><br><br><br><br>3 | 6<br>14<br><br>3<br><br>21<br>125<br>2<br>1<br>1<br><br>11<br>9<br>2<br>2<br>5<br>85<br><br><br>26<br><br><br><br>14 | <br>1<br><br><br><br><br>3<br><br><br><br><br><br><br><br><br><br>1<br><br><br><br><br><br><br> | | |

| | | | | | | | | | | |
|---|---|---|---|---|---|---|---|---|---|---|
| | **All Lamiales** | **24** | **1080** | **25494** | **366 (1.44%)** | **34** | **327** | **5** | **0.133%** | **9.3%** |
| [FFF] | Aquifoliales<br>[Eudicots – Asterids] | Aquifoliaceae [392] | 1 | 500 | 3 | | 3 | | | |
| | | Cardiopteridaceae [389] | 5 | 43 | | | | | | |
| | | Helwingiaceae [391] | 1 | 4 | | | | | | |
| | | Phyllonomaceae [390] | 1 | 4 | | | | | | |
| | | Stemonuraceae [388] | 12 | 90 | | | | | | |
| | **All Aquifoliales** | **5** | **20** | **641** | **3 (0.47%)** | | **3** | | **0%** | **0%** |
| [GGG] | Asterales<br>[Eudicots – Asterids] | Alseuosmiaceae [397] | 5 | 13 | | | | | | |
| | | Argophyllaceae [399] | 2 | 21 | 2 | | 2 | | | |
| | | Asteraceae [403] | 1623 | 24700 | 547 | 79 | 464 | 4 | | |
| | | Calyceraceae [402] | 4 | 60 | | | | | | |
| | | Campanulaceae [394] | 81 | 2800 | 87 | | 87 | | | |
| | | Goodeniaceae [401] | 12 | 440 | 6 | | 6 | | | |
| | | Menyanthaceae [400] | 6 | 60 | 2 | | 2 | | | |
| | | Pentaphragmataceae [395] | 1 | 30 | | | | | | |
| | | Phellinaceae [398] | 1 | 12 | | | | | | |
| | | Rousseaceae [393] | 4 | 6 | 1 | | 1 | | | |
| | | Stylidiaceae [396] | 6 | 245 | 2 | | 2 | | | |
| | **All Asterales** | **11** | **1745** | **28387** | **647 (2.28%)** | **79** | **564** | **4** | **0.278%** | **12.2%** |
| [HHH] | Escallioniales<br>[Eudicots – Asterids] | Escalloniaceae [404] | **7** | **103** | **1 (0.97%)** | | **1** | | **0%** | **0%** |
| [III] | Bruniales<br>[Eudicost – Asterids] | Bruniaceae [406] | 6 | 81 | 1 | | 1 | | | |
| | | Columelliaceae [405] | 2 | 8 | | | | | | |
| | **All Bruniales** | **2** | **8** | **89** | **1 (1.12%)** | | **1** | | **0%** | **0%** |
| [JJJ] | Paracryphiales<br>[Eudicots – Asterids] | Paracryphiaceae [407] | 3 | 36 | | | | | | **no data** |
| [KKK] | Dipsacales<br>[Eudicots – Asterids] | Adoxaceae [408] | 5 | 225 | 13 | 2 | 11 | | | |
| | | Caprifoliaceae [409] | 28 | 825 | 36 | 1 | 35 | | | |
| | **All Dipsacales** | **2** | **33** | **1050** | **49 (4.67%)** | **3** | **46** | | **0.286%** | **6.1%** |
| [LLL] | Apiales<br>[Eudicots – Asterids] | Apiaceae [416] | 442 | 3575 | 127 | 20 | 107 | | | |
| | | Araliaceae [414] | 43 | 1650 | 10 | | 10 | | | |
| | | Griseliniaceae [412] | 1 | 7 | | | | | | |
| | | Myodocarpaceae [415] | 2 | 15 | | | | | | |
| | | Pennantiaceae [410] | 1 | 4 | | | | | | |
| | | Pittosporaceae [413] | 7 | 245 | 11 | | 11 | | | |
| | | Torricelliaceae [411] | 3 | 10 | | | | | | |
| | **All Apiales** | **7** | **499** | **5506** | **148 (2.69%)** | **20** | **128** | | **0.363%** | **13.5%** |
| | **All Angiosperms** | **416** | **13164** | **295383** | **4052 (1.37%)** | **498** | **3525** | | **0.169%** | **12.3%** |

# Table S6: The Distribution of Ecdysteroid-Positive Species Within Angiosperm Orders; Data from the randomly selected samples (N-Series) of the Exeter Survey (ES)

Orders and Families in the angiosperms are listed according to APGIV (Bot J Linn Soc 181, 1-20, 2016). There are 64 Orders of angiosperms according to this classification. The numbers of genera and species in each family are taken from Christenhusz MJM & Byng JW (Phytotaxa 261(3), 201-217, 2016), which uses the classification of APGIV. The linear sequence of Orders is indicated by letters in square brackets ([A]→[Z]→[AA]→[ZZ]→[AAA]→[LLL]). The linear sequence of Families is indicated by numbers in square brackets ([1]→[416]). The Table is organised primarily according to the evolutionary sequence of the Orders, and secondarily alphabetically according to Family name, but the evolutionary sequence is provided by the Linear Sequence Numbers.

Data on the presence/absence of ecdysteroids derives from the Exeter Survey of plants for ecdysteroid agonists and antagonists (1995-2002; Version 1).

| Sequence | Angiosperm Order | Families + [linear sequence number] | No. Genera | No. Species | No. Species Assessed | No. Species Positive | No. Species Negative | No. Species Uncertain | % of All Species Positive | % of Assessed Species Positive |
|---|---|---|---|---|---|---|---|---|---|---|
| [A] | Amborellales [independent clade] | Amborellaceae [1] | 1 | 1 | | | | | | **no data** |
| [B] | Nympheales [Independent clade] | Cabombaceae [3]<br>Hydatellaceae [2]<br>Nymphaeaceae [4] | 2<br>1<br>5 | 6<br>12<br>70 | | | | | | |
| | **All Nympheales** | **3** | **8** | **88** | | | | | | **no data** |
| [C] | Austrobaileyales [Independent clade] | Austobaileyaceae [5]<br>Schisandraceae [7]<br>Trimeniaceae [6] | 1<br>3<br>1 | 1<br>85<br>8 | <br>2 | | <br>2 | | | |
| | **All Austrobaileyales** | **3** | **5** | **94** | **2 (2.128%)** | **0** | **2** | | **0%** | **0%** |
| [D] | Canellales [Magnolids] | Canellaceae [8]<br>Winteraceae [9] | 5<br>5 | 23<br>65 | <br>1 | | <br>1 | | | |
| | **All Canellales** | **2** | **10** | **88** | **1 (1.136%)** | | **1** | | **0%** | **0%** |
| [E] | Piperales [Mangolids] | Aristolochiaceae [12]<br>Piperaceae [11]<br>Saururaceae [10] | 7<br>5<br>4 | 500<br>3700<br>6 | 3<br>1 | 1 | 2<br>1 | | | |
| | **All Piperales** | **3** | **16** | **4206** | **4 (0.095%)** | **1** | **3** | | **0.024%** | **25.0%** |
| [F] | Magnoliales [Magnolids] | Annonaceae [18]<br>Degeneriaceae [15]<br>Eupomatiaceae [17]<br>Himantandraceae [16]<br>Magnoliaceae [14]<br>Myristicaceae [13] | 105<br>1<br>1<br>1<br>2<br>21 | 2500<br>2<br>3<br>2<br>294<br>520 | 2<br><br><br><br>4 | 1<br><br><br><br>2 | 1<br><br><br><br>2 | | | |

| | | | | | | | | | | |
|---|---|---|---|---|---|---|---|---|---|---|
| | **All Magnoliales** | **6** | **131** | **3321** | **6 (0.181%)** | **3** | **3** | | **0.090%** | **50.0%** |
| [G] | Laurales [Magnolids] | Anthereospermataceae [22] | 6 | 16 | | | | | | |
| | | Calycathaceae [19] | 3 | 10 | 2 | | 2 | | | |
| | | Gomortegaceae [21] | 1 | 1 | | | | | | |
| | | Hernandiaceae [23] | 5 | 58 | | | | | | |
| | | Lauraceae [25] | 45 | 2850 | 2 | | 2 | | | |
| | | Monimiaceae [24] | 24 | 217 | | | | | | |
| | | Siparunaceae [20] | 2 | 75 | | | | | | |
| | **All Laurales** | **7** | **86** | **3227** | **4 (0.124%)** | **0** | **4** | | **0%** | **0%** |
| [H] | Chloranthales [Independent clade] | Chloranthaceae [26] | 4 | 77 | | | | | | **no data** |
| [I] | Acorales [Monocots] | Acoraceae [27] | 1 | 2 | | | | | | **no data** |
| [J] | Alismatales [Monocots] | Alismataceae [30] | 17 | 115 | 3 | | 3 | | | |
| | | Aponogetonaceae [34] | 1 | 56 | | | | | | |
| | | Araceae [28] | 114 | 3750 | 13 | | 13 | | | |
| | | Butomaceae [31] | 1 | 1 | | | | | | |
| | | Cymodoceaceae [41] | 5 | 17 | | | | | | |
| | | Hydrocharitaceae [32] | 16 | 135 | | | | | | |
| | | Juncaginaceae [35] | 3 | 34 | | | | | | |
| | | Maundiaceae [36] | 1 | 1 | | | | | | |
| | | Posidoniaceae [39] | 1 | 9 | | | | | | |
| | | Potamogetonaceae [38] | 6 | 110 | | | | | | |
| | | Ruppiaceae [40] | 1 | 8 | | | | | | |
| | | Scheuchzeriaceae [33] | 1 | 1 | | | | | | |
| | | Tofieldiaceae [29] | 4 | 28 | | | | | | |
| | | Zosteraceae [37] | 2 | 22 | | | | | | |
| | **All Alismatales** | **14** | **173** | **4287** | **16** | **0** | **16** | | **0%** | **0%** |
| [K] | Petrosaviales [Monocots] | Petrosaviaceae [42] | 2 | 4 | | | | | | **no data** |
| [L] | Dioscoreales [Monocots] | Burmanniaceae [44] | 8 | 99 | | | | | | |
| | | Dioscoreaceae [45] | 9 | 715 | 1 | | 1 | | | |
| | | Nartheciaceae [43] | 5 | 38 | | | | | | |
| | **All Dioscoreales** | **3** | **22** | **852** | **1 (0.117%)** | **0** | **1** | | **0%** | **0%** |
| [M] | Pandanales [Monocots] | Cyclanthaceae [49] | 12 | 230 | | | | | | |
| | | Pandanaceae [50] | 5 | 982 | | | | | | |
| | | Stemonaceae [48] | 4 | 37 | | | | | | |
| | | Triuridaceae [46] | 9 | 55 | | | | | | |
| | | Velloziaceae [47] | 5 | 306 | | | | | | |
| | **All Pandanales** | **5** | **35** | **1610** | | | | | | **no data** |
| [N] | Liliales [Monocots] | Alstroemeriaceae [55] | 4 | 254 | 4 | | 4 | | | |
| | | Campynemataceae [51] | 2 | 4 | | | | | | |

| | | | | | | | | | | |
|---|---|---|---|---|---|---|---|---|---|---|
| | | Colchicaceae [56] | 15 | 285 | 5 | | 5 | | | |
| | | Corsiaceae [52] | 3 | 27 | | | | | | |
| | | Liliaceae [60] | 15 | 705 | 30 | 3 | 27 | | | |
| | | Melanthiaceae [53] | 17 | 173 | 3 | 3 | | | | |
| | | Petermanniaceae [54] | 1 | 1 | | | | | | |
| | | Philesiaceae [57] | 2 | 2 | | | | | | |
| | | Ripogonaceae [58] | 1 | 6 | | | | | | |
| | | Smilacaceae [59] | 1 | 255 | 1 | | 1 | | | |
| | **All Liliales** | **10** | **61** | **1712** | **43 (2.512%)** | **6** | **37** | | **0.351%** | **13.95%** |
| [O] | Asparagales [Monocots] | Amaryllidaceae [73] | 75 | 1600 | 29 | 4 | 25 | | | |
| | | Asparagaceae [74] | 114 | 2900 | 47 | 11 | 36 | | | |
| | | Asphodelaceae [72] | 39 | 900 | 10 | | 10 | | | |
| | | Asteliaceae [64] | 3 | 37 | | | | | | |
| | | Blandfordiaceae [63] | 1 | 4 | 1 | 1 | | | | |
| | | Boryaceae [62] | 2 | 12 | | | | | | |
| | | Doryanthaceae [67] | 1 | 2 | 1 | 1 | | | | |
| | | Hypoxidaceae [66] | 4 | 159 | 1 | 1 | | | | |
| | | Iridaceae [70] | 66 | 2244 | 31 | 2 | 29 | | | |
| | | Ixioliriaceae [68] | 1 | 4 | | | | | | |
| | | Lanariaceae [65] | 1 | 1 | | | | | | |
| | | Orchidaceae [61] | 736 | 28000 | 1 | | 1 | | | |
| | | Tecophilaeaceae [69] | 9 | 27 | | | | | | |
| | | Xeronemataceae [71] | 1 | 2 | 1 | | 1 | | | |
| | **All Asparagales** | **14** | **1053** | **35892** | **122 (0.340%)** | **20** | **102** | | **0.056%** | **16.39%** |
| [P] | Arecales [Monocots] | Arecaceae [76] | 181 | 2600 | 15 | 1 | 14 | | | |
| | | Dasypogonaceae [75] | 4 | 16 | | | | | | |
| | **All Arecales** | **2** | **185** | **2616** | **15 (0.578%)** | **1** | **14** | | **0.038%** | **6.67%** |
| [Q] | Commelinales [Monocots] | Commelinaceae [78] | 41 | 731 | 2 | 2 | | | | |
| | | Haemodoraceae [81] | 14 | 102 | 6 | | 6 | | | |
| | | Hanguanaceae [77] | 1 | 12 | | | | | | |
| | | Philydraceae [79] | 3 | 6 | | | | | | |
| | | Pontederiaceae [80] | 6 | 34 | | | | | | |
| | **All Commelinales** | **5** | **65** | **885** | **8 (0.904%)** | **2** | **6** | | **0.226%** | **25.0%** |
| [R] | Zingiberales [Monocots] | Cannaceae [86] | 1 | 10 | 3 | 1 | 2 | | | |
| | | Costaceae [88] | 7 | 143 | 1 | | 1 | | | |
| | | Heliconiaceae [84] | 1 | 194 | | | | | | |
| | | Lowiaceae [83] | 1 | 18 | | | | | | |
| | | Marantaceae [87] | 29 | 570 | | | | | | |
| | | Musaceae [85] | 3 | 91 | 3 | | 3 | | | |
| | | Strelitziaceae [82] | 3 | 7 | 3 | | 3 | | | |
| | | Zingiberaceae [89] | 50 | 1600 | 5 | | 5 | | | |
| | **All Zingiberales** | **8** | **95** | **2633** | **15 (0.570%)** | **1** | **14** | | **0.038%** | **6.67%** |

| | | | | | | | | | | |
|---|---|---|---|---|---|---|---|---|---|---|
| [S] | Poales [Monocots] | Bromeliaceae [91] | 51 | 3475 | 1 | | 1 | | | |
| | | Cyperaceae [98] | 9 | 5500 | 14 | 2 | 12 | | | |
| | | Ecdeiocoleaceae [102] | 2 | 3 | | | | | | |
| | | Eriocaulaceae [94] | 7 | 1207 | | | | | | |
| | | Flagellariaceae [100] | 1 | 4 | | | | | | |
| | | Joinvilleaceae [101] | 1 | 4 | | | | | | |
| | | Juncaceae [97] | 8 | 464 | 2 | | 2 | | | |
| | | Mayacaceae [95] | 1 | 6 | | | | | | |
| | | Poaceae [103] | 780 | 12000 | 38 | 6 | 32 | | | |
| | | Rapateaceae [92] | 16 | 94 | | | | | | |
| | | Restionaceae [99] | 51 | 572 | 2 | | 2 | | | |
| | | Thurniaceae [96] | 2 | 4 | | | | | | |
| | | Typhaceae [90] | 2 | 51 | 1 | | 1 | | | |
| | | Xyridaceae [93] | 5 | 399 | 1 | | 1 | | | |
| | **All Poales** | **15** | **936** | **23783** | **59 (0.248%)** | **8** | **51** | | **0.034%** | **13.56%** |
| [T] | Ceratophyllales [Sister of Eudicots] | Ceratophyllaceae [104] | 1 | 4 | | | | | | **no data** |
| [U] | Ranunculales [Eudicots] | Berberidaceae [110] | 14 | 700 | 15 | 1 | 14 | | | |
| | | Circaeasteraceae [107] | 2 | 2 | | | | | | |
| | | Eupteleaceae [105] | 1 | 2 | | | | | | |
| | | Lardizabalaceae [108] | 7 | 40 | 1 | | 1 | | | |
| | | Menispermaceae [109] | 68 | 440 | | | | | | |
| | | Papaveraceae [106] | 42 | 775 | 41 | 2 | 39 | | | |
| | | Ranunculaceae [111] | 43 | 2346 | 95 | 19 | 76 | | | |
| | **All Ranunculales** | **7** | **177** | **4305** | **152 (3.531%)** | **22** | **13** | | **0.511%** | **14.47%** |
| [V] | Proteales [Eudicots] | Nelumbonaceae [113] | 1 | 3 | | | | | | |
| | | Platanaceae [114] | 1 | 8 | | | | | | |
| | | Proteaceae [115] | 83 | 1660 | 30 | | 30 | | | |
| | | Sabiaceae [112] | 3 | 66 | | | | | | |
| | **All Proteales** | **4** | **88** | **1737** | **30 (1.727%)** | **0** | **30** | | **0%** | **0%** |
| [W] | Trochodendrales [Eudicots] | Trochodendraceae [116] | 2 | 2 | | | | | | **no data** |
| [X] | Buxales [Eudicots] | Buxaceae [117] | 6 | 123 | | | | | | **no data** |
| [Y] | Gunnerales | Gunneraceae [119] | 1 | 63 | 2 | | 2 | | | |
| | | Myrothamnaceae [118] | 1 | 2 | | | | | | |
| | **All Gunnerales** | **2** | **2** | **65** | **2 (3.077%)** | **0** | **2** | | **0%** | **0%** |
| [Z] | Dilleniales [Core Eudicots] | Dilleniaceae [120] | 11 | 430 | **1 (0.233%)** | | **1** | | **0%** | **0%** |
| [AA] | Saxifragales [Eudicots – | Altingiaceae [123] | 1 | 15 | | | | | | |
| | | Aphanopetalaceae [131] | 1 | 2 | | | | | | |

| | | | | | | | | | | |
|---|---|---|---|---|---|---|---|---|---|---|
| | Superrosids] | Cercidiphyllaceae [125]<br>Crassulaceae [130]<br>Cynomoriaceae [135]<br>Daphniphyllaceae [126]<br>Grossulariaceae [128]<br>Haloragaceae [134]<br>Hamamelidaceae [124]<br>Iteaceae [127]<br>Paeoniaceae [122]<br>Penthoraceae [133]<br>Peridiscaceae [121]<br>Saxifragaceae [129]<br>Tetracarpaeaceae [132] | 1<br>35<br>1<br>1<br>1<br>19<br>26<br>2<br>1<br>1<br>4<br>33<br>1 | 2<br>1400<br>2<br>30<br>150<br>145<br>86<br>18<br>33<br>2<br>12<br>640<br>1 | 1<br>17<br><br><br>2<br><br>4<br><br>5<br><br><br>30<br> | <br><br><br><br><br><br><br><br>1 | 1<br>17<br><br><br>2<br><br>4<br><br>4<br><br><br>30<br> | | | |
| | **All Saxifragales** | **15** | **128** | **2538** | **59 (2.325%)** | **1** | **58** | | **0.039%** | **1.70%** |
| [BB] | Vitales<br>[Eudicots – Rosids] | Vitaceae [136] | 14 | 910 | **8 (0.899%)** | **0** | **8** | | **0%** | **0%** |
| [CC] | Zygophyllales<br>[Eudicots – Rosids] | Krameriaceae [137]<br>Zygophyllaceae [138] | 1<br>22 | 18<br>285 | | | | | | |
| | **All Zygophyllales** | **2** | **23** | **303** | | | | | | **no data** |
| [DD] | Fabales<br>[Eudicots – Rosids] | Fabaceae [140]<br>Polygalaceae [142]<br>Quillajaceae [139]<br>Surianaceae [141] | 751<br>21<br>1<br>5 | 19500<br>900<br>3<br>8 | 138<br>1 | 1 | 137<br>1 | | | |
| | **All Fabales** | **4** | **778** | **20411** | **139 (0.681%)** | **1** | **138** | | **0.005%** | **0.72%** |
| [EE] | Rosales<br>[Eudicots – Rosids] | Barbeyaceae [144]<br>Cannabaceae [149]<br>Dirachmaceae [145]<br>Elaegnaceae [146]<br>Moraceae [150]<br>Rhamnaceae [147]<br>Rosaceae [143]<br>Ulmaceae [148]<br>Urticaceae [151] | 1<br>8<br>1<br>3<br>38<br>55<br>91<br>7<br>53 | 1<br>100<br>2<br>60<br>1180<br>950<br>2950<br>45<br>2625 | <br>2<br><br>4<br>9<br>10<br>138<br>2<br>1 | <br><br><br><br><br><br>2 | <br>2<br><br>4<br>9<br>10<br>136<br>2<br>1 | | | |
| | **All Rosales** | **9** | **257** | **7913** | **166 (2.098%)** | **2** | **164** | | **0.025%** | **1.21%** |
| [FF] | Fagales<br>[Eudicots – Rosids] | Betulaceae [158]<br>Casuarinaceae [156]<br>Fagaceae [153]<br>Juglandaceae [155]<br>Myricaceae [154]<br>Nothofagaceae [152]<br>Ticodendraceae [157] | 6<br>4<br>8<br>9<br>3<br>1<br>1 | 167<br>91<br>927<br>50<br>57<br>48<br>1 | 31<br>2<br>1<br><br>1<br>2 | 2 | 28<br>2<br>1<br><br>1<br>2 | 1 | | |

| | | | | | | | | | | |
|---|---|---|---|---|---|---|---|---|---|---|
| | **All Fagales** | **7** | **32** | **1341** | **37 (0.276%)** | **2** | **34** | **1** | **0.149%** | **5.41%** |
| [GG] | Cucurbitales [Eudicots – Rosids] | Anisophyllaceae [160] | 4 | 71 | | | | | | |
| | | Apodanthaceae [159] | 2 | 10 | | | | | | |
| | | Begoniaceae [166] | 2 | 1825 | 5 | | 5 | | | |
| | | Coriariaceae [162] | 1 | 14 | 1 | | 1 | | | |
| | | Corynocarpaceae [161] | 1 | 5 | | | | | | |
| | | Cucurbitaceae [163] | 95 | 965 | 4 | | 4 | | | |
| | | Datiscaceae [165] | 1 | 3 | | | | | | |
| | | Tetramelaceae [164] | 2 | 2 | | | | | | |
| | **All Cucurbitales** | **8** | **110** | **2895** | **10 (0.345%)** | **0** | **10** | | **0%** | **0%** |
| [HH] | Celastrales [Eudicots – Rosids] | Celastraceae [168] | 96 | 1350 | 9 | | 9 | | | |
| | | Lepidobotryaceae [167] | 2 | 2 | | | | | | |
| | **All Celastrales** | **2** | **98** | **1352** | **9 (0.666%)** | | **9** | | **0%** | **0%** |
| [II] | Oxalidales [Eudicots – Rosids] | Brunelliaceae [175] | 1 | 60 | | | | | | |
| | | Cephalotaceae [174] | 1 | 1 | | | | | | |
| | | Connaraceae [170] | 12 | 180 | | | | | | |
| | | Cunoniaceae [172] | 27 | 330 | 5 | | 5 | | | |
| | | Elaeocarpaceae [173] | 12 | 615 | 4 | 1 | 3 | | | |
| | | Huaceae [169] | 2 | 4 | | | | | | |
| | | Oxalidaceae [171] | 5 | 570 | | | | | | |
| | **All Oxalidales** | **7** | **60** | **1760** | **9 (0.511%)** | **1** | **8** | | **0.057%** | **11.11%** |
| [JJ] | Malpighiales [Eudicots – Rosids] | Achariaceae [199] | 32 | 155 | | | | | | |
| | | Balanopacaceae [193] | 1 | 9 | | | | | | |
| | | Bonnetiaceae [182] | 3 | 35 | | | | | | |
| | | Calophyllaceae [184] | 14 | 475 | | | | | | |
| | | Caryocaraceae [187] | 2 | 26 | | | | | | |
| | | Centroplacaceae [190] | 2 | 6 | | | | | | |
| | | Chrysobalanaceae [197] | 18 | 533 | | | | | | |
| | | Clusiaceae [183] | 13 | 750 | | | | | | |
| | | Ctenolophonaceae [178] | 1 | 2 | | | | | | |
| | | Dichapetalaceae [195] | 3 | 170 | | | | | | |
| | | Elatinaceae [191] | 2 | 35 | | | | | | |
| | | Erythroxylaceae [180] | 4 | 242 | | | | | | |
| | | Euphorbiaceae [207] | 209 | 6252 | 19 | | 19 | | | |
| | | Euphroniaceae [196] | 1 | 3 | | | | | | |
| | | Goupiaceae [201] | 1 | 2 | | | | | | |
| | | Humiriaceae [198] | 5 | 56 | | | | | | |
| | | Hypericaceae [186] | 6 | 590 | 11 | 1 | 10 | | | |
| | | Irvingiaceae [177] | 3 | 13 | | | | | | |
| | | Ixonanthaceae [209] | 3 | 17 | | | | | | |
| | | Lacistemataceae [203] | 2 | 14 | | | | | | |
| | | Linaceae [208] | 10 | 255 | 4 | | 4 | | | |
| | | Lophopyxidaceae [188] | 1 | 1 | | | | | | |

| | | | | | | | | | | |
|---|---|---|---|---|---|---|---|---|---|---|
| | | Malphigiaceae [192] | 73 | 1315 | 1 | | 1 | | | |
| | | Ochnaceae [181] | 32 | 550 | 1 | | 1 | | | |
| | | Pandaceae [176] | 3 | 17 | | | | | | |
| | | Passifloraceae [202] | 29 | 980 | 12 | | 12 | | | |
| | | Peraceae [205] | 5 | 127 | | | | | | |
| | | Phyllanthaceae [211] | 57 | 2050 | | | | | | |
| | | Picrodendraceae [210] | 25 | 96 | | | | | | |
| | | Podostemaceae [185] | 46 | 300 | | | | | | |
| | | Putranjivaceae [189] | 2 | 216 | | | | | | |
| | | Rafflesiaceae [206] | 3 | 25 | | | | | | |
| | | Rhizophoraceae [179] | 15 | 147 | | | | | | |
| | | Salicaceae [204] | 56 | 1220 | 1 | | 1 | | | |
| | | Trigoniaceae [194] | 5 | 28 | | | | | | |
| | | Violaceae [200] | 31 | 980 | 9 | | 9 | | | |
| | **All Malpighiales** | **36** | **717** | **17692** | **58 (0.328%)** | **1** | **57** | | **0.006%** | **1.72%** |
| [KK] | Geraniales [Eudicots – Rosids] | Francoaceae [213] | 8 | 37 | 1 | | 1 | | | |
| | | Geraniaceae [212] | 5 | 830 | 25 | | 25 | | | |
| | **All Gerianales** | **2** | **13** | **867** | **26 (3.000%)** | **0** | **26** | | **0%** | **0%** |
| [LL] | Myrtales [Eudicots – Rosids] | Alzateaceae [221] | 1 | 1 | | | | | | |
| | | Combretaceae [214] | 10 | 530 | | | | | | |
| | | Crypteroniaceae [220] | 3 | 13 | | | | | | |
| | | Lythraceae [215] | 27 | 620 | 4 | | 4 | | | |
| | | Melastomataceae [219] | 165 | 5115 | 3 | | 3 | | | |
| | | Myrtaceae [218] | 132 | 5950 | 83 | 1 | 82 | | | |
| | | Onagraceae [216] | 22 | 656 | 25 | | 20 | | | |
| | | Penaeaceae [222] | 9 | 32 | | | | | | |
| | | Vochysiaceae [217] | 7 | 217 | | | | | | |
| | **All Myrtales** | **9** | **376** | **13134** | **110 (0.838%)** | **1** | **109** | | **0.008%** | **0.91%** |
| [MM] | Crossosomatales [Eudicots – Rosids] | Asphloaceae [223] | 1 | 1 | | | | | | |
| | | Crossosomataceae [229] | 4 | 10 | | | | | | |
| | | Geissolomataceae [224] | 1 | 1 | | | | | | |
| | | Guamatelaceae [227] | 1 | 1 | | | | | | |
| | | Stachyuraceae [228] | 1 | 8 | | | | | | |
| | | Staphyleaceae [226] | 2 | 45 | 1 | | 1 | | | |
| | | Strasburgeriaceae [225] | 2 | 2 | | | | | | |
| | **All Crossosomatales** | **7** | **12** | **68** | **1 (1.471%)** | **0** | **1** | | **0%** | **0%** |
| [NN] | Picramniales [Eudicots –Rosids] | Picramniaceae [230] | 3 | 49 | | | | | | **no data** |
| [OO] | Huerteales [Eudicots – Rosids] | Dipentodontaceae [234] | 2 | 20 | | | | | | |
| | | Gerrardinaceae [231] | 1 | 2 | | | | | | |
| | | Petenaeceae [232] | 1 | 1 | | | | | | |

| | | | | | | | | | | |
|---|---|---|---|---|---|---|---|---|---|---|
| | | Tapisciaceae [233] | 2 | 6 | | | | | | |
| | **All Huerteales** | **4** | **6** | **29** | | | | | | **no data** |
| [PP] | Sapindales | Anacardiaceae [239] | 83 | 860 | 11 | 1 | 10 | | | |
| | [Eudicots – Rosids] | Burseraceae [238] | 19 | 615 | | | | | | |
| | | Biebersteiniaceae [235] | 1 | 4 | | | | | | |
| | | Kirkiaceae [237] | 1 | 6 | | | | | | |
| | | Meliaceae [243] | 53 | 600 | 3 | 1 | 2 | | | |
| | | Nitrariaceae [236] | 3 | 19 | | | | | | |
| | | Rutaceae [241] | 148 | 2070 | 10 | 1 | 9 | | | |
| | | Sapindaceae [240] | 142 | 1860 | 26 | | 26 | | | |
| | | Simaroubaceae [242] | 22 | 108 | 1 | | 1 | | | |
| | **All Sapindales** | **9** | **472** | **6142** | **51 (0.830%)** | **3** | **48** | | **0.049%** | **5.88%** |
| [QQ] | Malvales | Bixaceae [250] | 4 | 23 | 2 | | 2 | | | |
| | [Eudicots –Rosids] | Cistaceae [251] | 9 | 170 | 13 | | 13 | | | |
| | | Cytinaceae [244] | 2 | 10 | | | | | | |
| | | Dipterocarpaceae [253] | 16 | 695 | | | | | | |
| | | Malvaceae [247] | 244 | 4225 | 34 | | 34 | | | |
| | | Muntingiaceae [245] | 3 | 3 | | | | | | |
| | | Neuradaceae [246] | 3 | 10 | | | | | | |
| | | Sarcolaenaceae [252] | 10 | 71 | | | | | | |
| | | Sphaerosepalaceae [248] | 2 | 18 | | | | | | |
| | | Thymelaeaceae [249] | 46 | 913 | 5 | 1 | 4 | | | |
| | **All Malvales** | **10** | **339** | **6138** | **54 (0.880%)** | **1** | **53** | | **0.016%** | **1.85%** |
| [RR] | Brassicales | Akaniaceae [254] | 2 | 2 | | | | | | |
| | [Eudicots – Rosids] | Bataceae [261] | 1 | 2 | | | | | | |
| | | Brassicaceae [270] | 328 | 3628 | 49 | 4 | 45 | | | |
| | | Capparaceae [268] | 30 | 324 | 1 | | 1 | | | |
| | | Caricaceae [257] | 6 | 35 | 3 | | 3 | | | |
| | | Cleomaceae [269] | 1 | 346 | 3 | | 3 | | | |
| | | Emblingiaceae [263] | 1 | 1 | | | | | | |
| | | Gyrostemonaceae [266] | 4 | 20 | | | | | | |
| | | Koeberliniaceae [260] | 1 | 2 | | | | | | |
| | | Limnanthaceae [258] | 2 | 8 | 1 | 1 | | | | |
| | | Moringaceae [256] | 1 | 13 | | | | | | |
| | | Pentadiplandraceae [265] | 1 | 1 | | | | | | |
| | | Resedaceae [267] | 12 | 107 | 2 | | 2 | | | |
| | | Salvadoraceae [262] | 3 | 11 | | | | | | |
| | | Setchellanthaceae [259] | 1 | 1 | | | | | | |
| | | Tovariaceae [264] | 1 | 2 | | | | | | |
| | | Tropaeolaceae [255] | 1 | 94 | 3 | | 3 | | | |
| | **All Brassicales** | **17** | **396** | **4596** | **62 (1.349%)** | **5** | **57** | | **0.109%** | **8.07%** |
| [SS] | Berberidopsidales | Aextoxicaceae [271] | 1 | 1 | | | | | | |
| | [Eudicots – | Berberidopsidaceae [272] | 2 | 3 | | | | | | |

| | | | | | | | | | | |
|---|---|---|---|---|---|---|---|---|---|---|
| | Superasterids] | | | | | | | | | |
| | **All Berberidopsidales** | **2** | **3** | **4** | | | | | | **no data** |
| [TT] | Santalales [Eudicots – Superasterids] | Balanophoraceae [275] | 17 | 44 | | | | | | |
| | | Loranthaceae [279] | 76 | 1050 | 1 | | 1 | | | |
| | | Misodendraceae [277] | 1 | 8 | | | | | | |
| | | Olacaceae [273] | 29 | 180 | | | | | | |
| | | Opiliaceae [274] | 11 | 33 | | | | | | |
| | | Santalaceae [276] | 43 | 1000 | 1 | | 1 | | | |
| | | Schoepfiaceae [278] | 3 | 58 | | | | | | |
| | **All Santalales** | **7** | **180** | **2373** | **2 (0.084%)** | **0** | **2** | | **0%** | **0%** |
| [UU] | Caryophyllales [Eudicots – Superasterids] | Achatocarpaceae [296] | 2 | 11 | | | | | | |
| | | Aizoaceae [304] | 121 | 1900 | 18 | | 18 | | | |
| | | Amaranthaceae [297] | 165 | 2040 | 14 | 5 | 9 | | | |
| | | Anacampserotaceae [316] | 3 | 36 | 1 | | 1 | | | |
| | | Ancistrocladaceae [288] | 1 | 21 | | | | | | |
| | | Asteropeiaceae [292] | 1 | 8 | | | | | | |
| | | Barbeuiaceae [302] | 1 | 1 | | | | | | |
| | | Basellaceae [312] | 4 | 19 | | | | | | |
| | | Cactaceae [317] | 127 | 1750 | 10 | | 10 | | | |
| | | Caryophyllaceae [295] | 81 | 2625 | 58 | 10 | 48 | | | |
| | | Didiereaceae [311] | 7 | 22 | | | | | | |
| | | Dioncophyllaceae [287] | 3 | 3 | | | | | | |
| | | Droseraceae [284] | 3 | 180 | 9 | | 9 | | | |
| | | Drosphyllaceae [286] | 1 | 1 | | | | | | |
| | | Frankeniaceae [280] | 1 | 90 | | | | | | |
| | | Gisekiaceae [303] | 1 | 8 | | | | | | |
| | | Halophytaceae [313] | 1 | 1 | | | | | | |
| | | Kewaceae [301] | 1 | 8 | | | | | | |
| | | Limeaceae [299] | 1 | 21 | | | | | | |
| | | Lophiocarpaceae [300] | 2 | 6 | | | | | | |
| | | Macarthuriaceae [293] | 1 | 10 | | | | | | |
| | | Microteaceae [294] | 1 | 9 | | | | | | |
| | | Molluginaceae [309] | 9 | 80 | | | | | | |
| | | Montiaceae [310] | 14 | 230 | 5 | | 5 | | | |
| | | Nepenthaceae [285] | 1 | 150 | | | | | | |
| | | Nyctaginaceae [308] | 31 | 400 | 1 | | 1 | | | |
| | | Petiveriaceae [306] | 9 | 20 | 2 | | 2 | | | |
| | | Physenaceae [291] | 1 | 2 | | | | | | |
| | | Phytolaccaceae [305] | 5 | 33 | 2 | | 2 | | | |
| | | Plumbaginaceae [282] | 30 | 725 | 9 | 1 | 8 | | | |
| | | Polygonaceae [283] | 48 | 1200 | 7 | | 7 | | | |

| | | | | | | | | | | |
|---|---|---|---|---|---|---|---|---|---|---|
| | | Portulacaceae [315] | 1 | 115 | 1 | | 1 | | | |
| | | Rhabdodendraceae [289] | 1 | 3 | | | | | | |
| | | Sarcobataceae [307] | 1 | 2 | | | | | | |
| | | Simmondsiaceae [290] | 1 | 1 | 1 | | 1 | | | |
| | | Stegnospermataceae [298] | 1 | 4 | | | | | | |
| | | Talinaceae [314] | 2 | 28 | 2 | | 2 | | | |
| | | Tamaricaceae [281] | 4 | 78 | | | | | | |
| | **All Caryophyllales** | **38** | **688** | **11841** | **140 (1.182%)** | **16** | **124** | | **0.135%** | **11.43%** |
| [VV] | Cornales [Eudicots – Asterids] | Cornaceae [324] | 2 | 85 | 10 | | 10 | | | |
| | | Curtisiaceae [322] | 1 | 1 | | | | | | |
| | | Grubbiaceae [323] | 1 | 3 | | | | | | |
| | | Hydrangeaceae [320] | 9 | 223 | 3 | | 3 | | | |
| | | Hydrostachyaceae [319] | 1 | 22 | | | | | | |
| | | Loasaceae [321] | 20 | 308 | 5 | 2 | 3 | | | |
| | | Nyssaceae [318] | 5 | 37 | 2 | 1 | 1 | | | |
| | **All Cornales** | **7** | **39** | **679** | **20 (2.946%)** | **3** | **17** | | **0.442%** | **15.0%** |
| [WW] | Ericales [Eudicots – Asterids] | Actinidiaceae [342] | 3 | 360 | 3 | | 3 | | | |
| | | Balsaminaceae [325] | 2 | 1000 | 1 | | 1 | | | |
| | | Clethraceae [343] | 2 | 75 | 1 | 1 | | | | |
| | | Cyrillaceae [344] | 2 | 2 | | | | | | |
| | | Diapensiaceae [338] | 5 | 12 | | | | | | |
| | | Ebenaceae [334] | 4 | 800 | 5 | 1 | 4 | | | |
| | | Ericaceae [345] | 124 | 4250 | 48 | 1 | 47 | | | |
| | | Fouquieriaceae [328] | 1 | 11 | | | | | | |
| | | Lecythidaceae [330] | 25 | 355 | 1 | | 1 | | | |
| | | Marcgraviaceae [326] | 7 | 120 | | | | | | |
| | | Mitrastemonaceae [346] | 1 | 2 | | | | | | |
| | | Pentaphylaceae [332] | 12 | 330 | | | | | | |
| | | Polemoniaceae [329] | 26 | 350 | 16 | 1 | 15 | | | |
| | | Primulaceae [335] | 53 | 2790 | 63 | 2 | 61 | | | |
| | | Roridulaceae [341] | 1 | 2 | | | | | | |
| | | Sapotaceae [333] | 54 | 1273 | | | | | | |
| | | Sarraceniaceae [340] | 3 | 34 | 1 | | 1 | | | |
| | | Sladeniaceae [331] | 2 | 3 | | | | | | |
| | | Styracaceae [339] | 11 | 160 | 1 | | 1 | | | |
| | | Symplocaceae [337] | 2 | 260 | | | | | | |
| | | Tetrameristaceae [327] | 3 | 5 | | | | | | |
| | | Theaceae [336] | 9 | 240 | 1 | | 1 | | | |
| | **All Ericales** | **22** | **352** | **12435** | **141 (1.134%)** | **6** | **135** | | **0.048%** | **4.26%** |
| [XX] | Icacinales | Icacinaceae [348] | 25 | 165 | | | | | | |

| | | | | | | | | | | |
|---|---|---|---|---|---|---|---|---|---|---|
| | [Eudicots – Asterids] | Oncothecaceae [347] | 1 | 2 | | | | | | |
| | **All Icacinalaes** | **2** | **26** | **167** | | | | | | **no data** |
| [YY] | Metteniusales<br>[Eudicots – Asterids] | Metteniusaceae [349] | 11 | 50 | | | | | | **no data** |
| [ZZ] | Garryales<br>[Eudicots – Asterids] | Eucommiaceae [350]<br>Garryaceae [351] | 1<br>2 | 1<br>25 | 1<br>2 | | 1<br>2 | | | |
| | **All Garryales** | **2** | **3** | **26** | **3 (11.539%)** | **0** | **3** | | **0%** | **0%** |
| [AAA] | Gentianales<br>[Eudicots – Asterids] | Apocynaceae [356]<br>Gelsemiaceae [355]<br>Gentianaceae [353]<br>Loganiaceae [354]<br>Rubiaceae [352] | 366<br>3<br>102<br>15<br>590 | 5100<br>11<br>1735<br>390<br>13620 | 16<br><br>27<br><br>14 | | 16<br><br>27<br><br>14 | | | |
| | **All Gentianales** | **5** | **1076** | **20856** | **57 (0.273%)** | **0** | **57** | | **0%** | **0%** |
| [BBB] | Boraginales<br>[Eudicots – Asterids] | Boraginaceae [357] | 135 | 2535 | **27<br>(0.1065%)** | **3** | **24** | | **0.118%** | **11.11%** |
| [CCC] | Vahliales<br>[Eudicots – Asterids] | Vahliaceae [358] | 1 | 8 | | | | | | **no data** |
| [DDD] | Solanales<br>[Eudicots – Asterids] | Convolvulaceae [359]<br>Hydroleaceae [363]<br>Montiniaceae [361]<br>Solanaceae [360]<br>Sphenocleaceae [362] | 53<br>1<br>3<br>100<br>1 | 1660<br>12<br>5<br>2600<br>2 | 8<br><br><br>25<br> | <br><br><br>2<br> | 8<br><br><br>23<br> | | | |
| | **All Solanales** | **5** | **158** | **4279** | **33 (0.771%)** | **2** | **31** | | **0.047%** | **6.06%** |
| [EEE] | Lamiales<br>[Eudicots – Asterids] | Acanthaceae [377]<br>Bignoniaceae [378]<br>Byblidaceae [374]<br>Calceolariaceae [368]<br>Carlemanniaceae [365]<br>Gesneriaceae [369]<br>Lamiaceae [383]<br>Lentibulariaceae [379]<br>Linderniaceae [373]<br>Martyniaceae [375]<br>Mazaceae [384]<br>Oleaceae [366]<br>Orobanchaceae [387]<br>Paulowniaceae [386]<br>Pedaliaceae [376]<br>Phyrmaceae [385]<br>Plantaginaceae [370]<br>Plocospermataceae [364]<br>Schlegeliaceae [380] | 210<br>82<br>1<br>2<br>2<br>152<br>241<br>3<br>23<br>5<br>3<br>26<br>98<br>3<br>13<br>13<br>94<br>1<br>4 | 4000<br>870<br>7<br>271<br>5<br>3540<br>7530<br>316<br>220<br>16<br>33<br>790<br>1960<br>8<br>75<br>136<br>1900<br>1<br>37 | 3<br>10<br><br>3<br><br>17<br>86<br>2<br>1<br>1<br><br>11<br>5<br>2<br><br>5<br>66<br><br> | <br><br><br><br><br>1<br>4<br><br><br><br><br><br><br><br><br><br>8<br><br> | 3<br>9<br><br>3<br><br>16<br>81<br>2<br>1<br>1<br><br>11<br>5<br>2<br><br>5<br>57<br><br> | <br>1<br><br><br><br><br>1<br><br><br><br><br><br><br><br><br><br>1<br><br> | | |

| | | | | | | | | | | |
|---|---|---|---|---|---|---|---|---|---|---|
| | | Scrophulariaceae [371]<br>Stilbaceae [372]<br>Tetrachondraceae [367]<br>Thomandersiaceae [381]<br>Verbenaceae [382] | 62<br>8<br>2<br>1<br>32 | 1830<br>40<br>3<br>6<br>1000 | 20<br><br><br><br>7 | 1 | 19<br><br><br><br>7 | | | |
| | **All Lamiales** | **24** | **1080** | **25494** | **239 (0.938%)** | **14** | **222** | **3** | **0.055%** | **5.86%** |
| [FFF] | Aquifoliales [Eudicots – Asterids] | Aquifoliaceae [392]<br>Cardiopteridaceae [389]<br>Helwingiaceae [391]<br>Phyllonomaceae [390]<br>Stemonuraceae [388] | 1<br>5<br>1<br>1<br>12 | 500<br>43<br>4<br>4<br>90 | 3 | | 3 | | | |
| | **All Aquifoliales** | **5** | **20** | **641** | **3 (0.468%)** | **0** | **3** | | **0%** | **0%** |
| [GGG] | Asterales [Eudicots – Asterids] | Alseuosmiaceae [397]<br>Argophyllaceae [399]<br>Asteraceae [403]<br>Calyceraceae [402]<br>Campanulaceae [394]<br>Goodeniaceae [401]<br>Menyanthaceae [400]<br>Pentaphragmataceae [395]<br>Phellinaceae [398]<br>Rousseaceae [393]<br>Stylidiaceae [396] | 5<br>2<br>1623<br>4<br>81<br>12<br>6<br>1<br>1<br>4<br>6 | 13<br>21<br>24700<br>60<br>2800<br>440<br>60<br>30<br>12<br>6<br>245 | <br>2<br>181<br><br>68<br>5<br>2<br><br><br>1<br>2 | <br><br>19 | <br>2<br>162<br><br>68<br>5<br>2<br><br><br>1<br>2 | | | |
| | **All Asterales** | **11** | **1745** | **28387** | **261 (0.919%)** | **19** | **242** | | **0.067%** | **7.28%** |
| [HHH] | Escallioniales [Eudicots – Asterids] | Escalloniaceae [404] | 7 | 103 | **1 (0.971%)** | | **1** | | **0%** | **0%** |
| [III] | Bruniales [Eudicost – Asterids] | Bruniaceae [406]<br>Columelliaceae [405] | 6<br>2 | 81<br>8 | 1 | | 1 | | | |
| | **All Bruniales** | **2** | **8** | **89** | **1** (1.124%) | | **1** | | 0% | 0% |
| [JJJ] | Paracryphiales [Eudicots – Asterids] | Paracryphiaceae [407] | 3 | 36 | | | | | | **no data** |
| [KKK] | Dipsacales [Eudicots – Asterids] | Adoxaceae [408]<br>Caprifoliaceae [409] | 5<br>28 | 225<br>825 | 8<br>25 | <br>1 | 8<br>24 | | | |
| | **All Dipsacales** | **2** | **33** | **1050** | **33 (3.143%)** | **1** | **32** | | **0.095%** | **3.03%** |
| [LLL] | Apiales [Eudicots – Asterids] | Apiaceae [416]<br>Araliaceae [414]<br>Griseliniaceae [412]<br>Myodocarpaceae [415]<br>Pennantiaceae [410] | 442<br>43<br>1<br>2<br>1 | 3575<br>1650<br>7<br>15<br>4 | 38<br>7 | 8 | 30<br>7 | | | |

| | | | | | | | | | | |
|---|---|---|---|---|---|---|---|---|---|---|
| | | Pittosporaceae [413]<br>Torricelliaceae [411] | 7<br>3 | 245<br>10 | 4 | | 4 | | | |
| | **All Apiales** | **7** | **499** | **5506** | **49 (0.890%)** | **8** | **41** | | **0.890%** | **16.33%** |
| | **All Angiosperms** | **416** | **13164** | **295383** | **2290 (0.775%)** | **154** | **2132** | **4** | **0.052%** | **6.73%** |
